# Nightside clouds and disequilibrium chemistry on the hot Jupiter WASP-43b

**DOI:** 10.1038/s41550-024-02230-x

**Published:** 2024-04-30

**Authors:** Taylor J. Bell, Nicolas Crouzet, Patricio E. Cubillos, Laura Kreidberg, Anjali A. A. Piette, Michael T. Roman, Joanna K. Barstow, Jasmina Blecic, Ludmila Carone, Louis-Philippe Coulombe, Elsa Ducrot, Mark Hammond, João M. Mendonça, Julianne I. Moses, Vivien Parmentier, Kevin B. Stevenson, Lucas Teinturier, Michael Zhang, Natalie M. Batalha, Jacob L. Bean, Björn Benneke, Benjamin Charnay, Katy L. Chubb, Brice-Olivier Demory, Peter Gao, Elspeth K. H. Lee, Mercedes López-Morales, Giuseppe Morello, Emily Rauscher, David K. Sing, Xianyu Tan, Olivia Venot, Hannah R. Wakeford, Keshav Aggarwal, Eva-Maria Ahrer, Munazza K. Alam, Robin Baeyens, David Barrado, Claudio Caceres, Aarynn L. Carter, Sarah L. Casewell, Ryan C. Challener, Ian J. M. Crossfield, Leen Decin, Jean-Michel Désert, Ian Dobbs-Dixon, Achrène Dyrek, Néstor Espinoza, Adina D. Feinstein, Neale P. Gibson, Joseph Harrington, Christiane Helling, Renyu Hu, Nicolas Iro, Eliza M.-R. Kempton, Sarah Kendrew, Thaddeus D. Komacek, Jessica Krick, Pierre-Olivier Lagage, Jérémy Leconte, Monika Lendl, Neil T. Lewis, Joshua D. Lothringer, Isaac Malsky, Luigi Mancini, Megan Mansfield, Nathan J. Mayne, Thomas M. Evans-Soma, Karan Molaverdikhani, Nikolay K. Nikolov, Matthew C. Nixon, Enric Palle, Dominique J. M. Petit dit de la Roche, Caroline Piaulet, Diana Powell, Benjamin V. Rackham, Aaron D. Schneider, Maria E. Steinrueck, Jake Taylor, Luis Welbanks, Sergei N. Yurchenko, Xi Zhang, Sebastian Zieba

**Affiliations:** 1 BAER Institute, NASA Ames Research Center, Moffet Field, CA USA; 2 Space Science and Astrobiology Division, NASA Ames Research Center, Moffett Field, CA USA; 3 Leiden Observatory, University of Leiden, Leiden, The Netherlands; 4 INAF - Osservatorio Astrofisico di Torino, Turin, Italy; 5 Space Research Institute, Austrian Academy of Sciences, Graz, Austria; 6 Max Planck Institute for Astronomy, Heidelberg, Germany; 7 Earth and Planets Laboratory, Carnegie Institution for Science, Washington DC, USA; 8 School of Physics and Astronomy, University of Leicester, Leicester, UK; 9 Universidad Adolfo Ibáñez: Peñalolén, Santiago, Chile; 10 School of Physical Sciences, The Open University, Milton Keynes, UK; 11 Department of Physics, New York University Abu Dhabi, Abu Dhabi, United Arab Emirates; 12 Center for Astro, Particle and Planetary Physics (CAP3), New York University Abu Dhabi, Abu Dhabi, United Arab Emirates; 13 Department of Physics and Trottier Institute for Research on Exoplanets, Université de Montréal, Montreal, Quebec Canada; 14 Université Paris-Saclay, Université Paris Cité, CEA, CNRS, AIM, Gif-sur-Yvette, France; 15 Atmospheric, Oceanic and Planetary Physics, Department of Physics, University of Oxford, Oxford, UK; 16 DTU Space, Technical University of Denmark, Kongens Lyngby, Denmark; 17 Space Science Institute, Boulder, CO USA; 18 Université Côte d’Azur, Observatoire de la Côte d’Azur, CNRS Laboratoire Lagrange, Nice, France; 19 Johns Hopkins APL, Laurel, MD USA; 20 LESIA, Observatoire de Paris, Université PSL, Sorbonne Université, Université Paris Cité, CNRS, Meudon, France; 21 Laboratoire de Météorologie Dynamique, IPSL, CNRS, Sorbonne Université, Ecole Normale Supérieure, Université PSL, Ecole Polytechnique, Institut Polytechnique de Paris, Paris, France; 22 Department of Astronomy and Astrophysics, University of Chicago, Chicago, IL USA; 23 Department of Astronomy and Astrophysics, University of California, Santa Cruz, Santa Cruz, CA USA; 24 Centre for Exoplanet Science, University of St Andrews, St Andrews, UK; 25 Center for Space and Habitability, University of Bern, Bern, Switzerland; 26 Space and Planetary Sciences, Institute of Physics, University of Bern, Bern, Switzerland; 27 Center for Astrophysics | Harvard & Smithsonian, Cambridge, MA USA; 28 Instituto de Astrofsica de Canarias (IAC), Tenerife, Spain; 29 INAF- Palermo Astronomical Observatory, Piazza del Parlamento, Palermo, Italy; 30 Department of Space, Earth and Environment, Chalmers University of Technology, Gothenburg, Sweden; 31 Department of Astronomy, University of Michigan, Ann Arbor, MI USA; 32 Department of Earth and Planetary Sciences, Johns Hopkins University, Baltimore, MD USA; 33 Department of Physics and Astronomy, Johns Hopkins University, Baltimore, MD USA; 34 Tsung-Dao Lee Institute, Shanghai Jiao Tong University, Shanghai, People’s Republic of China; 35 School of Physics and Astronomy, Shanghai Jiao Tong University, Shanghai, People’s Republic of China; 36 Université Paris Cité and Univ Paris Est Creteil, CNRS, LISA, Paris, France; 37 School of Physics, University of Bristol, Bristol, UK; 38 Indian Institute of Technology, Indore, India; 39 Centre for Exoplanets and Habitability, University of Warwick, Coventry, UK; 40 Department of Physics, University of Warwick, Coventry, UK; 41 Anton Pannekoek Institute for Astronomy, University of Amsterdam, Amsterdam, The Netherlands; 42 Departamento de Astrofsica, Centro de Astrobiologa (CAB, CSIC-INTA), ESAC campus, Madrid, Spain; 43 Instituto de Astrofisica, Facultad Ciencias Exactas, Universidad Andres Bello, Santiago, Chile; 44 Centro de Astrofisica y Tecnologias Afines (CATA), Santiago, Chile; 45 Nucleo Milenio de Formacion Planetaria (NPF), Valparaíso, Chile; 46 Department of Physics and Astronomy, University of Kansas, Lawrence, KS USA; 47 Institute of Astronomy, Department of Physics and Astronomy, KU Leuven, Leuven, Belgium; 48 Space Telescope Science Institute, Baltimore, MD USA; 49 Department of Astrophysical and Planetary Sciences, University of Colorado Boulder, Boulder, CO USA; 50 School of Physics, Trinity College Dublin, Dublin, Ireland; 51 Planetary Sciences Group, Department of Physics and Florida Space Institute, University of Central Florida, Orlando, FL USA; 52 Astrophysics Section, Jet Propulsion Laboratory, California Institute of Technology, Pasadena, CA USA; 53 Division of Geological and Planetary Sciences, California Institute of Technology, Pasadena, CA USA; 54 Institute of Planetary Research—Extrasolar Planets And Atmospheres, German Aerospace Center (DLR), Berlin, Germany; 55 Department of Astronomy, University of Maryland, College Park, MD USA; 56 European Space Agency, Space Telescope Science Institute, Baltimore, MD USA; 57 California Institute of Technology, IPAC, Pasadena, CA USA; 58 Laboratoire d’Astrophysique de Bordeaux, Université de Bordeaux, Pessac, France; 59 Département d’Astronomie, Université de Genève, Sauverny, Switzerland; 60 Department of Mathematics and Statistics, University of Exeter, Exeter, UK; 61 Department of Physics, Utah Valley University, Orem, UT USA; 62 Department of Physics, University of Rome “Tor Vergata”, Rome, Italy; 63 INAF - Turin Astrophysical Observatory, Turin, Italy; 64 Steward Observatory, University of Arizona, Tucson, AZ USA; 65 Department of Physics and Astronomy, Faculty of Environment, Science and Economy, University of Exeter, Exeter, UK; 66 School of Information and Physical Sciences, University of Newcastle, Callaghan, NSW Australia; 67 Universitäts-Sternwarte, Ludwig-Maximilians-Universität München, Munich, Germany; 68 Exzellenzcluster Origins, Garching, Germany; 69 Department of Earth, Atmospheric and Planetary Sciences, Massachusetts Institute of Technology, Cambridge, MA USA; 70 Kavli Institute for Astrophysics and Space Research, Massachusetts Institute of Technology, Cambridge, MA USA; 71 Centre for ExoLife Sciences, Niels Bohr Institute, Copenhagen, Denmark; 72 School of Earth and Space Exploration, Arizona State University, Tempe, AZ USA; 73 Department of Physics and Astronomy, University College London, London, UK; 74 Department of Earth and Planetary Sciences, University of California, Santa Cruz, Santa Cruz, CA USA

**Keywords:** Exoplanets, Exoplanets

## Abstract

Hot Jupiters are among the best-studied exoplanets, but it is still poorly understood how their chemical composition and cloud properties vary with longitude. Theoretical models predict that clouds may condense on the nightside and that molecular abundances can be driven out of equilibrium by zonal winds. Here we report a phase-resolved emission spectrum of the hot Jupiter WASP-43b measured from 5 μm to 12 μm with the JWST’s Mid-Infrared Instrument. The spectra reveal a large day–night temperature contrast (with average brightness temperatures of 1,524 ± 35 K and 863 ± 23 K, respectively) and evidence for water absorption at all orbital phases. Comparisons with three-dimensional atmospheric models show that both the phase-curve shape and emission spectra strongly suggest the presence of nightside clouds that become optically thick to thermal emission at pressures greater than ~100 mbar. The dayside is consistent with a cloudless atmosphere above the mid-infrared photosphere. Contrary to expectations from equilibrium chemistry but consistent with disequilibrium kinetics models, methane is not detected on the nightside (2*σ* upper limit of 1–6 ppm, depending on model assumptions). Our results provide strong evidence that the atmosphere of WASP-43b is shaped by disequilibrium processes and provide new insights into the properties of the planet’s nightside clouds. However, the remaining discrepancies between our observations and our predictive atmospheric models emphasize the importance of further exploring the effects of clouds and disequilibrium chemistry in numerical models.

## Abstract (web summary)

Phase-resolved mid-infrared observations from JWST of the hot gas giant WASP-43b detect a day–night difference of 659 ± 19 K. Comparison with climate models shows that the observations are compatible with cloudy skies, at least on the nightside, and the lack of methane detection suggests the presence of disequilibrium chemistry.

## Main

Hot Jupiters are tidally synchronized to their host stars, with vast differences in irradiation between the dayside and nightside. Previous observations with the Hubble Space Telescope (HST) and the Spitzer Space Telescope show that these planets have cooler nightsides and weaker hotspot offsets than expected from cloud-free three-dimensional models^1–5^. The main mechanism believed to be responsible for this behaviour is the presence of nightside clouds, which would hide the thermal flux of the planet and lead to a sharp longitudinal gradient in brightness temperature^3,4,6–10^. Other mechanisms have been proposed, such as the presence of atmospheric drag due to hydrodynamic instabilities or magnetic coupling^11–13^, super-stellar atmospheric metallicity^14,15^, or interaction between the deep winds and the photosphere^16^, but these mechanisms are less universal than the cloud hypothesis^17,18^.

WASP-43b, a hot Jupiter with an orbital period of just 19.5 h (ref. ^19^), is an ideal target for thermal phase-curve observations. Its host star is a K7 main-sequence star 87 pc away with metallicity close to solar and weak variability^20^. Previous measurements of the planet’s orbital phase curve in the near-infrared have revealed a large temperature contrast between the dayside and nightside hemispheres, broadly consistent with the presence of nightside clouds^3,21,22^, which could be composed of magnesium silicates (Mg_2_SiO_4_/MgSiO_2_) and other minerals (for example, MnS, Na_2_S, metal oxides)^23,24^. Owing to the low nightside flux, the exact temperature and cloud properties were challenging to determine from previous observations^4,25,26^. With the mid-infrared capabilities of the JWST, we have the opportunity to measure the phase-resolved thermal spectrum with unprecedented sensitivity, particularly on the cold nightside. We observed a full orbit of WASP-43b in the 5–12 μm range with the JWST’s Mid-Infrared Instrument (MIRI)^27^ in low-resolution spectroscopy (LRS)^28^ slitless mode on 1 and 2 December 2022, as part of the Transiting Exoplanet Community Early Release Science Program (JWST-ERS-1366). This continuous observation lasted 26.5 h at a cadence of 10.34 s (9,216 integrations) and included a full phase curve with one transit and two eclipses.

## Results

**Fig. 1:**
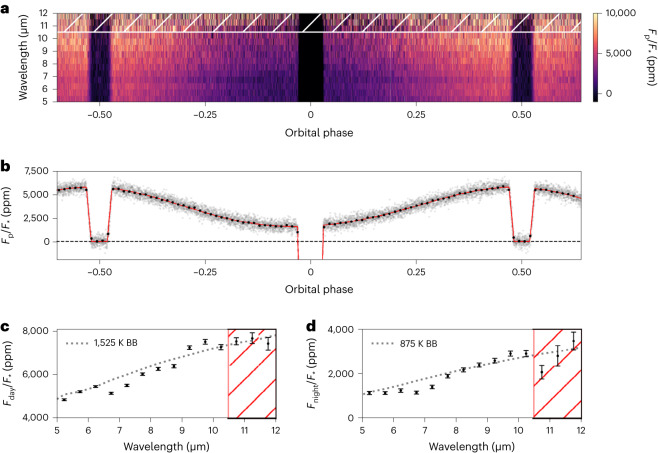
A visualization of the observed light curves and the resulting emission spectra. **a**, The observed spectroscopic light curves binned to a 0.5 μm wavelength resolution and after systematic noise removal, following the Eureka! v1 methods. The first 779 integrations have been removed from this figure and our fits as they were impacted by strongly decreasing flux. Wavelengths longer than 10.5 μm marked with a hatched region were affected by the ‘shadowed region effect’ (Methods) and could not be reliably reduced. **b**, The observed band-integrated light curve after systematic noise removal (grey points) and binned data with a cadence of 15 min (black points, with error bars smaller than the point sizes), compared with the best-fitting astrophysical model (red line). **c**,**d**, The measured dayside (**c**) and nightside (**d**) emission spectra are shown with black points and 1*σ* error bars, and black-body curves (dotted line denoted as ‘BB’, assuming a PHOENIX^74–76^ model for the star) are shown to emphasize planetary spectral features with black-body temperatures estimated by eye to match the continuum flux levels. Wavelengths longer than 10.5 μm were affected by the shadowed region effect and are unreliable.

**Extended Data Fig. 1:**
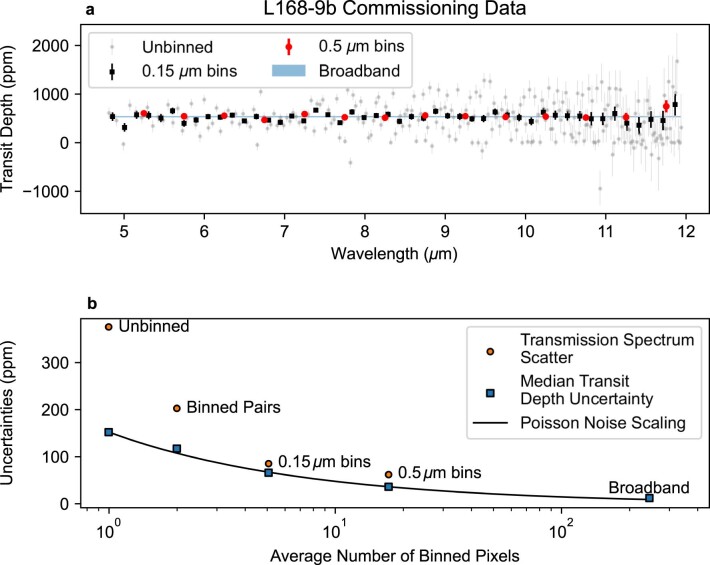
The underestimation of uncertainties as a function of spectral binning for the L168-9b commissioning observations. **a**, The observed L168-9b transmission spectrum with 1*σ* error bars for spectrally unbinned data (grey circles), 0.15 *μ*m bins (black squares), 0.5 *μ*m bins (large red circles), and a 5-12 *μ*m broadband bin (horizontal blue shaded region). The spectrum for wavelength pairs is not shown to avoid excessive clutter. **b**, The median of the transit depth uncertainties are shown with blue squares, while the observed scatter in the transmission spectrum is shown with orange circles. For unbinned data, the transmission spectrum shows about 2.5 × the scatter predicted by the fits to the individual light curves. Binning pairs of wavelengths reduces the level of underestimation of the scatter in the transmission spectrum, but considerable excess noise remains. Coarser binning schemes like the constant 0.15 *μ*m bins used in the MIRI time-series observation commissioning paper^29^ or the 0.5 *μ*m bins we use in this work further reduce the level of uncertainty underestimation.

**Extended Data Fig. 2:**
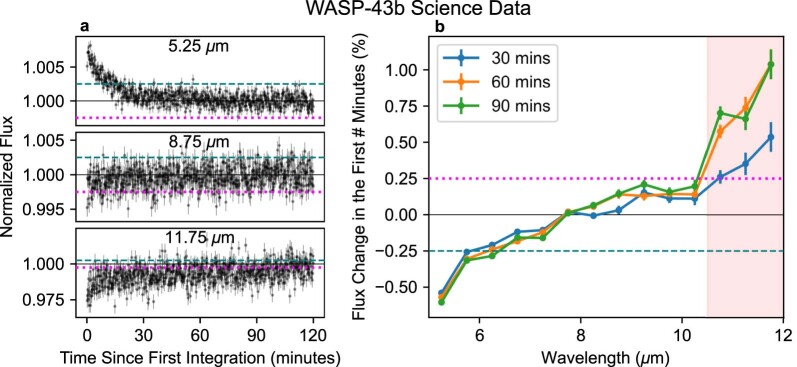
A model-independent demonstration of the initial changes in flux for the WASP-43b observations. **a**, The first 120 minutes of three of our spectroscopically binned light curves of WASP-43b (with 1*σ* uncertainties) showing the initial settling behaviour as a function of wavelength. A teal dashed line shows the amplitude of a -0.25% change in flux compared to the values around 120 minutes, and a magenta dotted line shows a +0.25% change. **b**, A summary of the ramp amplitudes, signs, and timescales for each of our wavelength bins (with 1*σ* uncertainties). The teal and magenta horizontal lines are the same as those in panel a to aid in translating between the two figures. At short wavelengths, the flux sharply drops by about 0.5% within the first 30 minutes and then largely settles but does continue to decrease with time. With increasing wavelength, the strength of this initial ramp decreases and eventually changes sign, becoming an upwards ramp. Within the ‘shadowed region’ (marked in red), the light curves show a very strong upwards ramp that takes much longer (greater than about 60 minutes) to appreciably decay. It is important to note that the data in this figure also includes a small amount of astrophysical phase variations which should result in a small increase in flux of less than 0.05% per hour.

We performed multiple independent reductions and fits to these observations (see ‘Data reduction pipelines’ and ‘Light-curve fitting’ in Methods) to ensure robust conclusions. Our analyses all identified a strong systematic noise feature from 10.6 μm to 11.8 μm, the source of which is still unclear, and we were unable to adequately detrend these 10.6–11.8 μm data (see ‘Shadowed region effect’ in Methods). As shown in Extended Data Fig. 1, we also found that larger wavelength bins were required to accurately estimate our final spectral uncertainties (see ‘Spectral binning’ in Methods). As a result, our final analyses consider only the 5–10.5 μm data, which we split into 11 channels with a constant 0.5 μm wavelength spacing. Similar to the MIRI commissioning time-series observations, our data show a strong downwards exponential ramp in the first ~60 min and a weaker ramp throughout the observation^29^ (Extended Data Fig. 2). To minimize correlations with the phase variations, we removed the initial strong ramp by excluding the first 779 integrations (134.2 min) and then fitted a single exponential ramp model to the remaining data. A single ramp effectively removed the systematic noise, with the broadband light curve showing scatter ~1.25× the expected photon noise, while the spectroscopic light curves reach as low as ~1.1× the photon limit, probably due to improved decorrelation of wavelength-dependent systematics. Figure 1 shows the spectral light curves, broadband light curve, dayside spectra and nightside spectra from our fiducial reduction and fit.

From our Eureka! v1 analysis (Methods), we measure a broadband (5–10.5 μm) peak-to-trough phase variation of 4,180 ± 33 ppm with an eclipse depth of 5,752 ± 19 ppm and a nightside flux of 1,636 ± 37 ppm. Assuming a PHOENIX stellar model and marginalizing over the published stellar and system parameters^30^, the broadband dayside brightness temperature is 1,524 ± 35 K while the nightside is 863 ± 23 K. This corresponds to a day–night brightness temperature contrast of 659 ± 19 K, in agreement with the large contrasts previously observed^4,21,22,25^. The phase variations are well fitted by a sum of two sinusoids (the first and second harmonics), with two sinusoids preferred over a single sinusoid at 16*σ* (see ‘Determining the number of sinusoid harmonics’ in Methods) for the broadband light curve. The peak brightness of the broadband phase curve occurs at 7.34 ± 0.38° E from the substellar point (although individual reductions find offsets ranging from 7.34° E to 9.60° E), while previous studies have found offsets of 12.3 ± 1.0° E for HST Wide Field Camera 3’s (WFC3) 1.1–1.7 μm bandpass^21^, offsets ranging from 4.4° E to 12.2° E for Spitzer InfraRed Array Camera’s (IRAC) 3.6 μm filter^22,25^ and offsets ranging from 10.4° E to 21.1° E for Spitzer/IRAC’s 4.5 μm filter^4,22,25,26,31,32^. Overall, these broadband data represent roughly an order of magnitude in improved precision on the eclipse depth (6×), phase-curve amplitude (6×) and phase-curve offset (10×) over individual Spitzer/IRAC 4.5 μm observations of the system^22,26,32^; this improvement is largely driven by the JWST’s larger mirror (45×), about 12× less pointing jitter (per axis), about 4× improved stability in the width of the point spread function (PSF) along each axis and MIRI’s much broader bandpass.

## Model interpretation

To interpret the measurements, we compared the observations with synthetic phase curves and emission spectra derived from general circulation models (GCMs). Simulations were gathered from five different modelling groups, amounting to 31 separate GCM realizations exploring a range of approaches and assumptions. Notably, in addition to cloud-free simulations, the majority of the GCMs modelled clouds with spatial distributions that were either fully predicted^5,26,33^ or simply limited to the planet’s nightside^4^. For the predictive cloud models, simulations favoured warmer, clearer daysides with cooler, cloudier nightsides, but the precise distributions varied with assumptions regarding cloud physics and compositions. In general, models with smaller cloud particles or extended vertical distributions tended to produce thicker clouds at the pressures sensed by the observations. Details of the different models are provided in Methods.

**Fig. 2:**
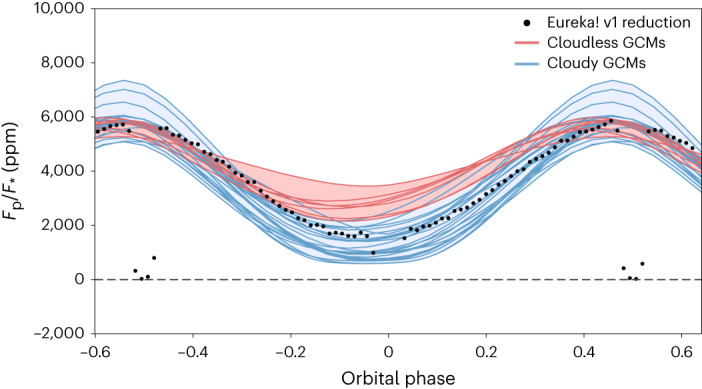
A comparison of the observed 5–10.5 μm light curve with GCM simulations. The black points show the temporally binned broadband light curve. The solid lines represent modelled phase curves derived from the 31 GCM simulations, integrated over the same wavelength range as the data, and separated into two groups based on the inclusion of clouds. The cloudless GCMs (red lines) simulated completely cloud-free skies, whereas the cloudy GCMs (blue lines) included at least some clouds on the nightside of the planet. The red and blue shaded areas span the range of all the cloudless and cloudy simulations, respectively, with the spread of values owing to differences in the various model assumptions and parameterizations. On average, the cloudless GCM phase curves have a maximum planet-to-star flux ratio of 5,703 ppm and a minimum of 2,681 ppm. This matches the observed maximum of the phase curve well but does not match its observed minimum at 1,636 ± 37 ppm. On average, the cloudy GCM phase curves have a maximum of 5,866 ppm and a minimum of 1,201 ppm, in better agreement with the observed nightside emission, but their spread of maximum values is much larger than the cloudless simulations. The cloudy models are able to suppress the nightside emission and better match the data; however, not all cloud models fit equally well and those with the optically thickest nightside clouds suppress too much emission. The models do not include the eclipse signals (phases −0.5 and 0.5) or transit signal (phase 0.0).

Despite fundamental differences in the models and the parameterizations they employ, simulated phase curves derived from models that include cloud opacity on the planet’s nightside provide a better match to the observed nightside flux compared with the clear simulations (Fig. 2). In contrast, the observed dayside fluxes (180° orbital phase) were matched similarly well by models with and without clouds. This implies the presence of widespread clouds preferentially on the planet’s nightside with cloud optical thicknesses sufficient to suppress thermal emission and cool the thermal photosphere. Specifically, models with integrated mid-infrared cloud opacities of roughly 2–4 above the 300 mbar level (that is, blocking ~87–98% of the underlying emission), best match the observed nightside flux.

Including nightside clouds also improved the agreement with the measured hotspot offset (7.34± 0.38° E). While cloudless models all produced eastward offsets greater than 16.6° (25.5° on average), simulations with clouds had offsets as low as 7.6° (with a mean of 16.4°). These reduced offsets were associated with decreases in the eastwards jet speeds of up to several kilometres per second, with maximum winds of roughly 2.0–2.5 km s^−1^ providing the best match (see Extended Data Table 1 for further details). This modelled jet-speed reduction is probably due to a disruption in the equatorwards momentum transport^34^ brought about by nightside clouds^4,35,36^. However, the resulting range of offsets seen in the suite of models suggests that this mechanism is quite sensitive to the details of cloud models, and other modelling factors (for example, atmospheric drag^11,12,16^, radiative timescales^14,15,37^) probably still play an important role.

**Fig. 3:**
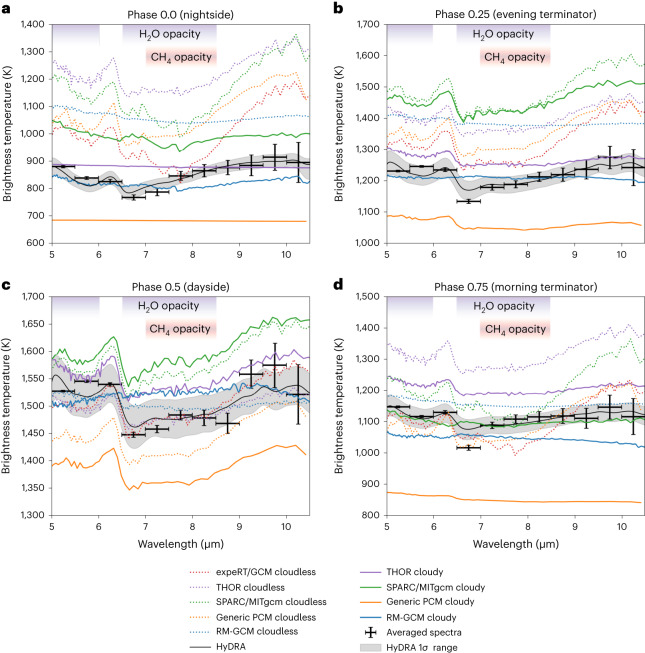
A comparison of the observed and modelled spectra at different phases. **a**–**c**, The observed emission spectrum with 1*σ* error bars at phases 0.0 (**a**), 0.25 (**b**), 0.5 (**c**) and 0.75 (**d**), along with select modelled spectra derived from different cloudy and cloudless GCMs (described in Methods and listed in Extended Data Table 1). Although absolute brightness temperatures differ appreciably between models owing to various GCM assumptions, differences in the relative shape of the spectra are strongly dependent on the cloud and temperature structure found in the GCMs (Extended Data Fig. 7). Models with more isothermal profiles (like RM-GCM) or thick clouds at pressures of ≲10–100 mbar (like THOR cloudy, Generic PCM with 0.1 μm cloud particles) produce flatter spectra, while clearer skies yield stronger absorption features. The observed spectra from the nightside and terminators appear muted compared with the clear-model spectra, suggesting the presence of at least some clouds or weak vertical temperature gradients at pressures of ≲10–100 mbar. In contrast, the spectral structure produced by water vapour opacity (indicated by the purple shading) appears more consistent with models lacking clouds at these low pressures on the dayside. Under equilibrium chemistry, methane would also show an absorption feature at ~7.5–8.5 μm (shaded pink) for the colder models at phases 0.0 and 0.75. Finally, the median retrieved spectrum and 1*σ* contours from the HyDRA retrieval are shown in grey.

A comparison of the observed and modelled emission spectra further suggests that the majority of the cloud thermal opacity must be confined to pressures greater than ~10–100 mbar, because the presence of substantial cloud opacity at lower pressures dampens the modelled spectral signature amplitude below what is observed (Fig. 3). No distinct spectral signatures indicative of the cloud composition were evident in the observations. While no single GCM can match the emission spectra at all phases, spectra corresponding to nightside, morning and evening terminators appear qualitatively similar to GCM results that are intermediate between clear and cloudy simulations. In contrast, the absorption features indicative of water vapour (between ~5 μm and 8.5 μm) seen in the dayside emission spectrum are more consistent with an absence of cloud opacity at these mid-infrared wavelengths. Altogether, these findings represent new constraints on the spatial distribution and opacity of WASP-43b’s clouds.

**Fig. 4:**
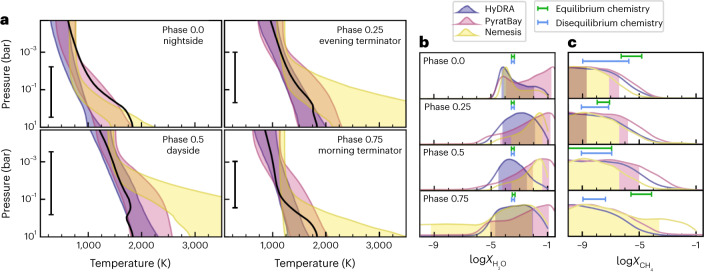
Atmospheric spectral retrievals for frameworks with free chemistry. **a**, Temperature profile contours (68% confidence) constrained by the retrievals at each orbital phase (see legends). All frameworks produced consistent non-inverted thermal profiles that are consistent with two-dimensional radiative–convective equilibrium and photochemical models along the equator^23^ (black curves) over the range of pressures probed by the observations (black bars). **b**, H_2_O abundance posterior distributions (volume mixing ratios). The shaded areas denote the span of the 68% confidence intervals. The green and blue bars on each panel denote the abundances predicted by equilibrium and disequilibrium chemistry solar-abundance models^23^, respectively, at the pressures probed by the observations (1–10^−3^ bar, approximately). **c**, The same as in **b** but for CH_4_. The retrieved water abundances are consistent with either equilibrium or disequilibrium chemistry estimations for solar composition (500 ppm), whereas the retrieved upper limits to the CH_4_ abundance are more consistent with disequilibrium chemistry predictions.

**Extended Data Fig. 3:**
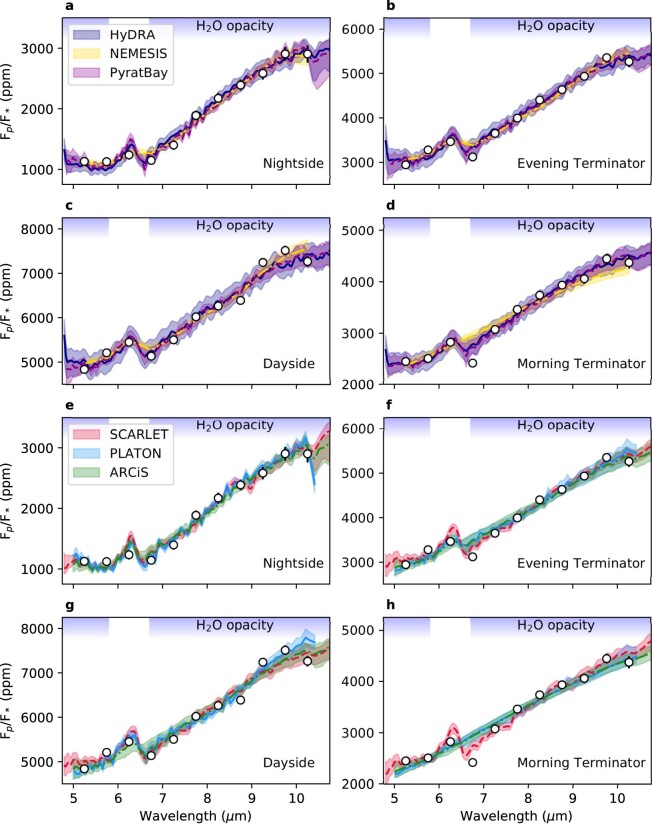
Retrieved spectra from the six retrievals. **a**, Median retrieved nightside spectra for the HyDRA (dark blue line), NEMESIS (dash-dotted gold line), and PyratBay (dashed magenta line) and their 1*σ* contour. The regions of higher water opacity are indicated by the purple shading at the top of the panel, with the observed rise in flux at 6.3 *μ*m being caused by a drop in opacity. **b**, **c**, and **d**, Same as panel a for the evening terminator, dayside, and morning terminator respectively. **e**, **f**, **g**, and **h**, Same as panels a, b, c, and d, for the SCARLET (dashed red line), PLATON (blue line), and ARCiS (dash-dotted green line) retrievals.

**Extended Data Fig. 4:**
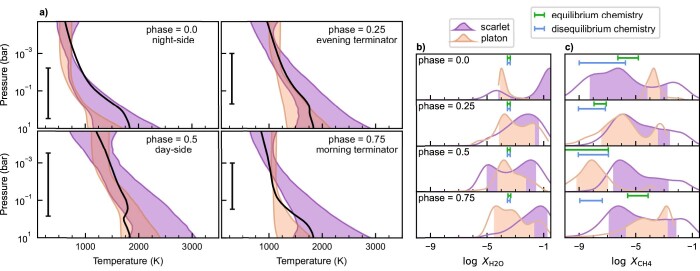
Chemically-consistent atmospheric retrievals. Same as Figure 4 but for retrievals assuming thermochemical-equilibrium abundances consistent with the pressure-temperature profiles. **a**, 1*σ* credible interval contours of the temperature profiles. The black curves show the predicted temperature profile from a 2D radiative-transport model46. The vertical bars show the range of pressures probed by the observations. **b** and **c**, probability posterior distributions for H_2_O and CH_4_ abundances, respectively. The shaded area for each curve denotes the 1*σ* credible interval of each posterior. The green and blue bars denote the abundances predicted by equilibrium and disequilibrium-chemistry models with solar abundances, respectively, at the pressures probed by the observations. Compared to the free-chemistry retrievals, the thermochemical-equilibrium retrievals on the nightside spectra produced worse fits, this is driven particularly by the higher amount of methane expected under equilibrium chemistry.

We further characterized the chemical composition of WASP-43b’s atmosphere by applying a suite of atmospheric retrieval frameworks to the phase-resolved emission spectra. The retrievals spanned a broad range of model assumptions, including free chemical abundances versus equilibrium chemistry, different temperature profile parameterizations and different cloud models (see ‘Atmospheric retrieval models’ in Methods). Despite these differences, the retrievals yielded consistent results for both the chemical and thermal constraints. We detected water vapour across the dayside, nightside, morning and evening hemispheres, with detection significances of up to ~3–4*σ* (Extended Data Fig. 3 and Extended Data Tables 2 and 3). The retrieved abundances of H_2_O largely lie in the 10–10^5^ ppm range for all four phases and for all the retrieval frameworks (Fig. 4 and Extended Data Fig. 4), broadly consistent with the value expected for a solar composition (500 ppm) as well as previous observations^22^.

**Extended Data Fig. 5:**
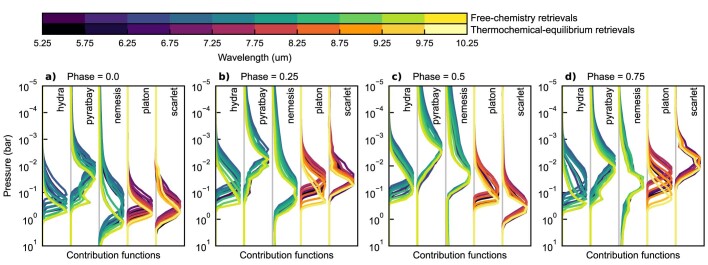
Retrieval contribution functions. Contribution functions integrated over the data point spectral bins, at each phase (**a-d**), and for each retrieval framework. These curves show the range of pressures probed by the observation according to the atmospheric models. The enhanced opacity from the water band around 7-9 *μ*m makes these wavelengths probe lower pressures and hence colder temperatures, whereas the rest of the observing window probes higher pressures and higher temperatures.

We also searched for signatures of disequilibrium chemistry in the atmosphere of WASP-43b. While CH_4_ is expected to be present on the nightside under thermochemical equilibrium conditions, we did not detect CH_4_ at any phase (Fig. 4). In the pressure range probed by the nightside spectrum (1–10^−3^ bar; Extended Data Fig. 5), the equilibrium abundance of CH_4_ is expected to vary between ~1 ppm and 100 ppm for a solar C/O ratio^23^, compared with our 95% upper limits of 1–6 ppm (Extended Data Table 2). The upper limits we place on the nightside CH_4_ abundance are more consistent with disequilibrium models that account for vertical and horizontal transport^23,24,38^. In particular, two-dimensional photochemical models and GCMs predict the strongest depletion of CH_4_ on the nightside due to strong zonal winds (>1 km s^−1^) transporting gas-phase constituents around the planet faster than the chemical reactions can maintain thermochemical equilibrium, thus ‘quenching’ and homogenizing the global composition at values more representative of dayside conditions (see also refs. ^39–42^). We note, however, that a low atmospheric C/O ratio and/or clouds at photospheric pressures could also lead to a non-detection of CH_4_. We also searched for signatures of NH_3_, which is predicted to have a volume mixing ratio less than 0.1–1 ppm in both equilibrium and disequilibrium chemistry models, and find that the results are inconclusive and model-dependent with the current retrieval frameworks.

**Extended Data Fig. 6:**
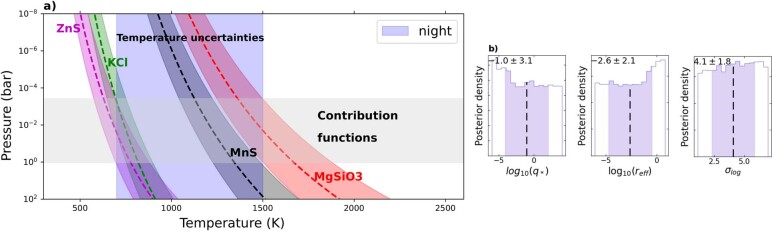
PyratBay clouds exploration. **a**, Cloud species that condense in the temperature regime expected for the WASP-43b nightside. Dashed lines represent vapour pressure curves^162^ for each species assuming solar composition, while the coloured ranges denote the corresponding extent of the vapour pressure curves assuming 100 × sub- and super-solar atmospheric composition. The extent of the retrieved nightside contribution functions is shown in grey, and the extent of the retrieved temperature uncertainties is shown in light purple. The intersection between the contribution function and temperature ranges indicates the pressures at which we could observe cloud condensation and potentially detect their spectral features, if present in the observations. **b**, Panels display the retrieved posterior density plots for the explored cloud parameters of the TSC model (cloud number density, q*; effective particle size, r_eff_; and the standard deviation of the log-normal distribution, ${\sigma }_{\log }$) for the MnS clouds. The black vertical line denotes the parameter’s median value, while the extent of the purple region denotes the 1*σ* uncertainties, both given at the top left corner of the panel. Similar, fully non-constrained posteriors are retrieved for other explored cloud species, MgSiO_3_, ZnS, and KCl, suggesting the lack of observable spectral characteristics from clouds in the observed data.

**Extended Data Fig. 7:**
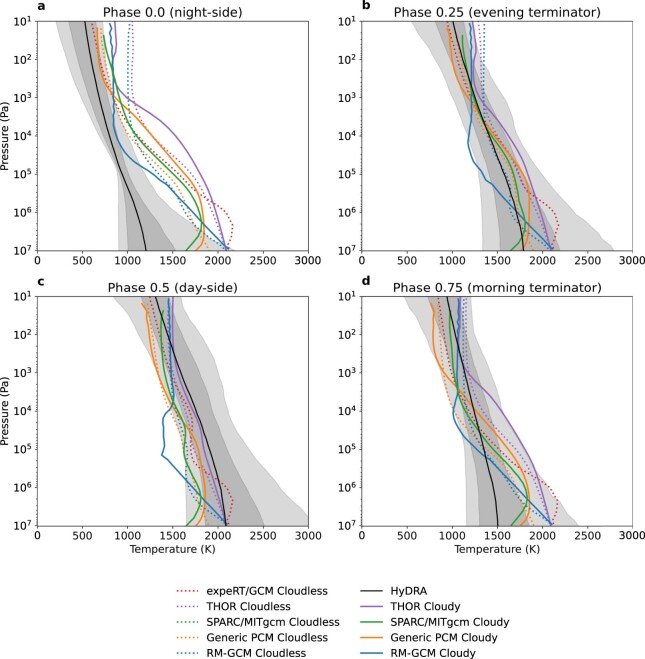
A comparison of the retrieved temperature-pressure profiles to the GCM simulations. Each of **a-d** shows the temperature profile retrieved by HyDRA, compared to the GCM simulations highlighted in Figure 3 and listed in Extended Data Table 1. The GCM temperature profiles are calculated at phases 0.0, 0.25, 0.5, and 0.75 by averaging over the visible hemisphere by viewing angle, to produce a one-dimensional profile that is comparable to the retrieved profile. The GCM simulations are generally warmer on the nightside than the retrieved temperatures; cloudy simulations emit from lower pressures and so match the observed lower brightness temperatures better (see the contribution functions in Extended Data Fig. 5).

Given the strong evidence for clouds from comparison with GCMs, we also searched for signatures of clouds in the atmospheric retrieval. Formally, the retrievals do not detect clouds with statistical significance, indicating that strong spectral features uniquely attributable to condensates are not visible in the data (see ‘Atmospheric retrieval models’ in Methods and Extended Data Fig. 6). However, the retrievals may mimic the effects of cloud opacity with a more isothermal temperature profile, as both tend to decrease the amplitude of spectral features, but the cloud-free, more isothermal temperature profile requires fewer free parameters and is therefore statistically favoured. Indeed, while the retrieved temperature profiles on the dayside and evening hemispheres agree well with the hemispherically averaged temperature profiles from the GCMs, they are more isothermal than the GCM predictions for the nightside and morning hemispheres (Extended Data Fig. 7). This discrepancy may hint at the presence of clouds on the nightside and morning hemispheres, consistent with the locations of clouds found in the GCMs.

Taken together, our results highlight the unique capabilities of JWST/MIRI for exoplanet atmosphere characterization. Combined with a range of atmospheric models, the observed phase curve and emission spectra provide strong evidence that the atmospheric chemistry of WASP-43b is shaped by complex disequilibrium processes and provide new constraints on the optical thickness and pressure of nightside clouds. However, while cloudy GCM predictions match the data better than cloud-free models, none of the simulations simultaneously reproduced the observed phase curve and spectra within measured uncertainties. These remaining discrepancies underscore the importance of further exploring the effects of clouds and disequilibrium chemistry in numerical models, as JWST continues to place unprecedented observational constraints on smaller and cooler planets.

## Methods

### Observations and quality of the data

We observed a full orbit of WASP-43b with the JWST MIRI LRS slitless mode as a part of JWST-ERS-1366. We performed target acquisition with the F1500W filter and used the SLITLESSPRISM subarray for the science observation. The science observation was taken between 1 December 2022 at 00:54:30 UT and 2 December 2022 at 03:23:36 UT, for a total of 26.5 h. We acquired 9,216 integrations, which were split into 3 exposures and 10 segments per exposure. Each integration lasts 10.34 s and is composed of 64 groups, with 1 frame per group. The LRS slitless mode reads an array of 416 × 72 pixels on the detector (the SLITLESSPRISM subarray) and uses the FASTR1 readout mode, which introduces an additional reset between integrations.

Owing to the long duration of the observation, two high-gain antenna moves occurred 8.828 h and 17.661 h after the start of the science observation. They affect only a couple of integrations that we removed from the light curves. A cross-shaped artefact is present on the two-dimensional images at the short-wavelength end due to light scattered by detector pixels^43^. It is stable over the duration of the observation but it contaminates the background and the spectral trace up to ~6 μm. This ‘cruciform’ artefact is observed in all MIRI LRS observations; a dedicated analysis is underway to estimate and mitigate its impact.

In the broadband light curve, the flux decays by ~0.1% during the first 60 min and continues to decay throughout the observation. This ramp is well modelled with 1 or 2 exponential functions after trimming the initial ~780 integrations. Without trimming any data, at least two ramps are needed. In addition, a downwards linear trend in flux is observed over the whole observation with a slope of −39 ppm per hour. These two types of drift also appear in the spectroscopic light curves. The exponential ramp amplitude in the first 60 min changes with wavelength from −0.67% in the 5–5.5 μm bin (downwards ramp) to +0.26% in the 10–10.5 μm bin (upwards ramp). The ramp becomes upwards at wavelengths longer than 7.5 μm and its timescale increases to more than 1 h at wavelengths longer than 10.5 μm. The slopes as a function of wavelength vary from −16 ppm to −52 ppm, all downwards. Such drifts (initial ramp and linear or polynomial trend) are also observed in other MIRI LRS time-series observations^29^ but the strength of the trends differ for each observation. In these WASP-43b observations, we note that their characteristic parameters vary smoothly with wavelength, which may help identify their cause and build correction functions.

Over the course of the observation, the position of the spectral trace on the detector varies by 0.0036 pixels RMS (0.027 pixels peak to peak) in the spatial direction, and the Gaussian standard deviation of the spatial PSF varies by 0.00069 pixels RMS (root mean square; 0.0084 pixels peak to peak) following a sharp increase by 0.022 pixels during the first 600 integrations. Depending on the wavelength bin, that spatial drift causes noise at the level of 7–156 ppm, while variations in the PSF width cause noise at the level of 4–54 ppm (these numbers are obtained from a linear decorrelation). Overall, the MIRI instrument used in LRS slitless mode remains remarkably stable over this 26.5-h-long continuous observation and the data are of exquisite quality.

The noise in the light curve increases sharply at wavelengths beyond 10.5 μm and the transit depths obtained at these long wavelengths by different reduction pipelines are discrepant. These wavelengths were not used in the retrieval analyses and the final broadband light curve. The cause is unknown but it might be related to the fact that this region of the detector receives different illumination before the observation^44^ (see ‘Shadowed region effect’ below for more details).

### Data reduction pipelines

#### Eureka! v1 reduction

The Eureka! v1 reduction made use of version 0.9 of the Eureka! pipeline^45^, CRDS version 11.16.16 and context 1018, and jwst package version 1.8.3^46^. The gain value of 5.5 electrons per data number obtained from these CRDS reference files is known to be incorrect, and the actual gain is estimated to be ~3.1 electrons per data number although the gain may be wavelength dependent (S. Kendrew, private communication). A new reference file reflecting the updated gain is under development at STScI, which will improve the accuracy of photon-noise calculations. For the rest of this analysis, we assume a constant gain of 3.1 electrons per data number. The Eureka! control files and Eureka! parameter files files used in these analyses are available for download (10.5281/zenodo.10525170) and are summarized below.

Eureka! makes use of the jwst pipeline for stages 1 and 2, and both stages were run with their default settings, with the exception of increasing the stage 1 jump step’s rejection threshold to 8.0 and skipping the photom step in stage 2 because it is not necessary and can introduce additional noise for relative time-series observations. In stage 3 of Eureka!, we then rotated the MIRI/LRS slitless spectra 90° anticlockwise so that wavelength increases from left to right like the other JWST instruments to allow for easier reuse of Eureka! functions. We then extracted pixels 11–61 in the new *y* direction (the spatial direction) and 140–393 in the new *x* direction (spectral direction); pixels outside of these ranges primarily contain noise that is not useful for our reduction. Pixels marked as ‘DO_NOT_USE’ in the DQ array were then masked as were any other unflagged NaN or inf pixels. A centroid was then fit to each integration by summing along the spectral direction and fitting the resulting one-dimensional profile with a Gaussian function; the centroid from the first integration was used for determining aperture locations, while the centroids and PSF widths from all integrations were saved to be used as covariates when fitting the observations.

Our background subtraction method is tailored to mitigate several systematic effects unique to the MIRI instrument. First, MIRI/LRS observations exhibit a ‘cruciform artefact’^43^ at short wavelengths caused by scattered light within the optics; this causes bright rays of scattered light which must be sigma-clipped to avoid over-subtracting the background. In addition, MIRI/LRS observations show periodic noise in the background flux, which drifts with time^29^ as well as 1/*f* noise^47^, which leads to correlated noise in the cross-dispersion direction; as a result, background subtraction must be performed independently for each integration and column (row in MIRI’s rotated reference frame). Furthermore, in both these observations and the dedicated background calibration observations from JWST-COM/MIRI-1053, we found that there was a linear trend in the background flux, with the background flux increasing with increasing row index (column index in MIRI’s rotated reference frame). To robustly remove this feature, we found that it was important to either (1) use the mean from an equal number of pixels on either side of the spectral trace for each column and integration, or (2) use a linear background model for each column and integration; we adopted the former as it resulted in less noisy light curves. To summarize, for each column in each integration we subtracted the mean of the pixels separated by ≥11 pixels from the centre of the spectral trace after first masking 5*σ* outliers in that column.

To compute the spatial profile for the optimal extraction of the source flux, we calculated a median frame, sigma-clipping 5*σ* outliers along the time axis and smoothing along the spectral direction using a 7-pixel-wide boxcar filter. During optimal extraction, we only used the pixels within 5 pixels of the fitted centroid and masked pixels that were 10*σ* discrepant with the spatial profile. Background exclusion regions ranging from 9 to 13 pixels and source aperture regions ranging from 4 to 6 pixels were considered, but our values of 11 and 5 pixels were selected as they produced the lowest median absolute deviation light curves before fitting.

#### Eureka! v2 reduction

The Eureka! v2 reduction followed the same procedure as the Eureka! v1 reduction except for the following differences. First, this reduction made use of version 1.8.1 of the jwst pipeline. For stage 1, we instead used a cosmic ray detection threshold of 5 and used a uniform ramp fitting weighting. For stage 2, we performed background subtraction using columns away from the trace on the left and on the right and subtracted the background for each integration^29^. Stage 3 was identical to Eureka! v1 reduction.

#### TEATRO reduction

We processed the data using the Transiting Exoplanet Atmosphere Tool for Reduction of Observations (TEATRO) that runs the jwst package, extracts and cleans the stellar spectra and light curves, and runs light-curve fits. We used the jwst package version 1.8.4, CRDS version 11.16.14 and context 1019. We started from the ‘uncal’ files and ran stages 1 and 2 of the pipeline. For stage 1, we set a jump rejection threshold of 6, turned off the ‘jump.flag_4_neighbors’ parameter and used the default values for all other parameters. For stage 2, we ran only the ‘AssignWcsStep’, ‘FlatFieldStep’ and ‘SourceTypeStep’; no photometric calibration was applied. The next steps were made using our own routines. We computed the background using two rectangular regions, one on each side of the spectral trace, between pixels 13 and 27 and between pixels 53 and 72 in the spatial direction, respectively. We computed the background value for each row (rows are along the spatial direction) in each region using a biweight location, averaged the two values and subtracted it from the full row. This background subtraction was done for each integration. Then, we extracted the stellar flux using aperture photometry by averaging pixels between 33 and 42 in each row to obtain the stellar spectrum at each integration. We also averaged pixels between 33 and 42 in the spatial direction and between 5 μm and 10.5 μm in the spectral direction to obtain the broadband flux. We averaged the spectra in 11 0.5-μm-wide wavelength channels. For each channel and for the broadband light curve, we normalized the light curve using the second eclipse as a reference flux, computed a running median filter using a 100-point window size, and rejected points that were more than 3*σ* away from that median using a 5-iteration sigma-clipping. To limit the impact of the initial ramp on the fitting, we trim the first 779 integrations from the broadband light curve and a similar number of integrations for each channel (the exact number depends on the channel). Finally, we subtracted 1 from the normalized light curves to have the secondary eclipse flux centred on 0. These cleaned light curves were used for phase curve, eclipse and transit fits.

#### SPARTA reduction

We reduced the data with the open-source Simple Planetary Atmosphere Reduction Tool for Anyone (SPARTA), first introduced in ref. ^48^ to analyse the MIRI LRS phase curve of GJ 1214b. We started from the uncalibrated data and proceeded all the way to the final results without using any code from the jwst or Eureka! pipelines. In stage 1, we started by discarding the first five groups as well as the last group, because these groups show anomalies due to the reset switch charge decay and the last-frame effect. We fitted a slope to the up-the-ramp reads in every pixel of every integration in every exposure. We calculated the residuals of these linear fits, and for every pixel, we computed a median residual for every group across all integrations. This ‘median residual’ array has dimensions *N*_grp_ × *N*_rows_ × *N*_cols_. This array was subtracted from the original uncalibrated data and the up-the-ramp fit was redone, this time without discarding any groups except those that were more than 5*σ* away from the best-fit line. Such outliers, which may be due to cosmic rays, were discarded and the fit recomputed until convergence. This procedure straightens out any nonlinearity in the up-the-ramp reads that is consistent across integrations, such as the reset switch charge decay, the last-frame effect or inaccuracies in the nonlinearity coefficients. After up-the-ramp fitting, we removed the background by removing the mean of columns 10–24 and 47–61 (inclusive, zero-indexed) for every row of every integration. As these two regions are of equal size and equally distant from the trace, any linear spatial trend in the background is naturally removed.

In the next step, we computed a pixel-wise median image over all integrations. This median image was used as a template to determine the position of the trace in each integration, by shifting and scaling the template until it matched the integration (and minimizes the *χ*^2^). It was also used as the point spread profile for optimal extraction, after shifting in the spatial direction by the amount calculated in the previous step. Outliers more than 5*σ* discrepant from the model image (which may be cosmic rays) were masked, and the optimal extraction was repeated until convergence. The *z*-scores image (image minus model image all divided by expected error, including photon noise and read noise) have a typical standard deviation of 0.88, compared with a theoretical minimum value of 1, indicating that the errors are being overestimated.

After optimal extraction, we gathered all the spectra and positions into one file. To reject outliers, we created a broadband light curve, detrended it by subtracting a median filter with a width 100 times less than the total data length and rejected integrations greater than 4*σ* away from 0 (which may be cosmic rays). Sometimes only certain wavelengths of an integration are bad, not the entire integration. We repaired these by detrending the light curve at each wavelength, identifying 4*σ* outliers and replacing them with the average of their two immediate temporal neighbours.

### Spectral binning

To investigate the effects of spectral binning, we utilized the MIRI time-series commissioning observations of the transit of L168-9b (JWST-COM/MIRI-1033; ref. ^29^). L168-9b was chosen to have a clear transit signal while also having no detectable atmospheric signatures expected in its mid-infrared transmission spectrum; as a result, the observed scatter in the transmission spectrum can be used as an independent measurement of the uncertainties in the transit depths. The same procedure cannot be done on our WASP-43b science observations as there may be detectable atmospheric signatures.

Following the Eureka! reduction methods described by ref. ^29^, we tried binning the L168-9b spectroscopic light curves at different resolutions and compared the observed standard deviation of the transmission spectrum with the median of the transit depth uncertainties estimated from fitting the spectral light curves. As shown in Extended Data Fig. 1, the uncertainties in the pixel-level light curves underestimate the scatter in the transmission spectrum by a factor of about two. Because pairs of rows (in MIRI’s rotated reference frame) are reset together, it is reasonable to assume that there could be odd–even effects that would average out if combining pairs of rows; indeed, there do appear to be differences in the amplitude of the initial exponential ramp feature between odd and even rows. However, combining pairs of rows still leads to appreciable underestimation of the scatter in the transmission spectrum. Interestingly, the underestimation of the uncertainties appears to decrease with decreasing wavelength resolution. This is likely explained by wavelength-correlated noise that gets averaged out with coarse binning. A likely culprit for this wavelength-correlated noise may be the 390 Hz periodic noise observed in several MIRI subarrays, which causes clearly structured noise with a period of ~9 rows^29^ (M. Ressler, private communication); this noise source is believed to be caused by MIRI’s electronics and possible mitigation strategies are still under investigation. Until the source of the excess wavelength-correlated noise is definitively determined and a noise mitigation method is developed, we recommend that MIRI/LRS observations should be binned to a fairly coarse spectral resolution as this gives better estimates of the uncertainties and also gives spectra that are closer to the photon-limited noise regime. However, we caution against quantitative extrapolations of the uncertainty underestimation to other datasets; because we do not know the source of the excess noise, we do not know how it might change with different parameters such as groups per integration or stellar magnitude.

Ultimately, for each reduction method, we binned the spectra down to a constant 0.50-μm-wavelength grid spanning 5–12 μm, giving a total of 14 spectral channels. However, as is described below, we only end up using the 11 spectral channels spanning 5–10.5 μm for science. This 0.5-μm-binning scheme combines 7 wavelengths for the shortest bin and 25 wavelengths for the longest bin, which has the added benefit of binning down the noise at longer wavelengths where there are fewer photons. However, even for this coarse of a binning scheme, we do expect there to be some additional noise beyond our estimated uncertainties on the spectrum of WASP-43b (Extended Data Fig. 1). As the structure of this noise source is not well understood nor is the extent to which our error bars are underestimated, our best course of action was to consider error inflation when performing spectroscopic inferences (described in more detail below).

### Light-curve fitting

#### Detrending the initial exponential ramp

As with other MIRI/LRS observations^29^, our spectroscopic light curves showed a strong exponential ramp at the start of the observations. As shown in Extended Data Fig. 2, the strength and sign of the ramp varies with wavelength, changing from a strong downwards ramp at 5 μm to a nearly flat trend around 8 μm, and then becoming an upwards ramp towards longer wavelengths. From 10.6 μm to 11.8 μm, the ramp timescale became much longer and the amplitude of the ramp became much stronger; this region of the detector was previously discussed^44^ and is discussed in more detail below. In general, most of the ramp’s strength had decayed within ~30–60 min, but at the precision of our data, the residual ramp signature still had an important impact on our nightside flux measurements due to the similarity of the ramp timescale with the orbital period. Unlike in the case of the MIRI/LRS commissioning observations of L168-9b^29^, we were not able to safely fit the entire dataset with a small number of exponential ramps. When fitting the entire dataset, we found that non-trivial choices about the priors for the ramp amplitudes and timescales resulted in significantly different spectra at phases 0.75 (morning hemisphere) and especially 0.0 (nightside); because the dayside spectrum is measured again near the end of the observations, it was less affected by this systematic noise.

Ultimately, we decided to conservatively discard the first 779 integrations (134.2 min), leaving only one transit duration of baseline before the first eclipse ingress began. After removing the initial 779 integrations, we found that a single exponential ramp model with broad priors that varied freely with wavelength was adequate to remove the signature. In particular, after removing the first 779 integrations we found that our dayside and nightside emission spectra were not significantly affected by (1) fitting two exponential ramps instead of one, (2) adjusting our priors on the ramp timescale to exclude rapidly decaying ramps with timescales >15 d^−1^ instead of >100 d^−1^, (3) putting a uniform prior on the inverse timescale instead of the timescale, or (4) altering the functional form of the ramp by fitting for an exponential to which the time was raised. After removing the first ~2 h, we also found that the ramp amplitude and timescale did not vary strongly with wavelength (excluding the ‘shadowed region’ described below), although fixing these parameters to those fitted to the broadband light curve affected several points in the nightside spectrum by more than 1*σ*; we ultimately decided to leave the timescale and amplitude to vary freely with wavelength as there is no a priori reason to assume that they should be equal. With careful crafting of priors, it appeared possible to get results similar to our final spectra while removing only the first few integrations, but trimming more integrations and only using a single exponential ramp model required fewer carefully tuned prior assumptions for which we have little physical motivation.

#### Shadowed region effect

As was described in ref. ^44^, we also identified a strong discontinuity in the spectroscopic light curves spanning pixel rows 156–220 (10.6–11.8 μm) in these observations. In this range, the temporal behaviour of the detector abruptly changes to a large-amplitude, long-timescale, upwards ramp that appears to slightly overshoot before decaying back down and approaching an equilibrium. These pixels coincide with a region of the detector between the Lyot coronagraph region and the four-quadrant phase mask region, which is unilluminated except when the dispersive element is in the optical path; as a result, we have taken to calling this unusual behaviour as the ‘shadowed region effect’. Strangely, not all MIRI/LRS observations show this behaviour, with the MIRI/LRS commissioning time-series observations^29^ and the GJ 1214b phase-curve observations^48^ showing no such effect. In fact, we know of only two other observations that show similar behaviour: the observation of the transit of WASP-80b (JWST-GTO-1177; T. Bell, private communication) and the observation of the phase curve of GJ 367b (JWST-GO-2508; M. Zhang, private communication). Informatively, the eclipse observation of WASP-80b taken 36 h after the WASP-80b transit using the same observing procedure (JWST-GTO-1177; T. Bell, private communication) did not show the same shadowed region effect, indicating that the effect is unlikely to be caused by stray light from nearby stars or any other factors that stayed the same between those two observations. Our best guess at this point is that the effect is related to the illumination history of the detector and the filter used by the previous MIRI observation (because the detector is illuminated at all times, even when it is not in use), but this is still under investigation and at present there is no way of predicting whether or not an observation will be impacted by the shadowed region effect. It is important to note, however, that from our limited knowledge at present that the shadowed region effect appears to be either present or not, with observations either strongly affected or seemingly completely unaffected.

Using the general methods described in the Eureka! v1 fit, we attempted to model the shadowed region effect with a combination of different ramp models, but nothing we tried was able to cleanly separate the effect from the phase variations, and there was always some clear structure left behind in the residuals of the fit. Another diagnostic that our detrending attempts were unsuccessful was that the phase offset as a function of wavelength smoothly varied around ~10° E in the unaffected region of the detector, but in the shadowed region, the phase offset would abruptly change to ~5° W; such a sharp change in a suspect region of the detector seems highly unlikely to be astrophysical in nature. As a result, we ultimately chose to exclude the three spectral bins spanning 10.5–12 μm from our retrieval efforts.

#### Determining the number of sinusoid harmonics

To determine the complexity of the phase-curve model required to fit the data, we used the Eureka! v1 reduction and most of the Eureka! v1 fitting methods described below, with the exception of using the dynesty^49^ nested sampling algorithm (which computes the Bayesian evidence, ${{{\mathcal{Z}}}}$) and a batman transit and eclipse model. Within dynesty, we used 256 live points, ‘multi’ bounds, ‘rwalk’ sampling, and ran until the estimated $d\ln ({{{\mathcal{Z}}}})$ reached 0.1. We then evaluated first-, second- and fourth-order models for the broadband light curve, excluding all third-order sinusoidal terms from the fourth-order model as these terms are not likely to be produced by the planet’s thermal radiation^50,51^. We then compared the Bayesian evidences of the different models following refs. ^52,53^ and found that the second-order model was significantly preferred over the first-order model at 16*σ* (${{\Delta }}\ln ({{{\mathcal{Z}}}})=128$), while the second-order model was insignificantly preferred over the fourth-order model at 2.2*σ* (${{\Delta }}\ln ({{{\mathcal{Z}}}})=1.3$). This is also confirmed by eye where the first-order model leaves clear phase-variation signatures in the residuals, while the residuals from the second-order model leave no noticeable phase variations behind. Finally, we also compared the phase-resolved spectra obtained from different order phase-curve models; we found that our spectra significantly changed going from a first- to second-order model (altering one or more spectral points by >1*σ*), but the fourth-order model did not significantly change the resulting phase-resolved spectra compared with the second-order. As a result, the final fits from all reductions used a second-order model. The broadband light curves obtained from the four reductions and the associated phase-curve models are shown in Supplementary Fig. 1.

#### Eureka! v1 fitting methods

We first sigma-clipped any data points that were 4*σ* discrepant from a smoothed version of the data (made using a boxcar filter with a width of 20 integrations) to remove any obviously errant data points while preserving the astrophysical signals like the transit.

Our astrophysical model consisted of a starry^54^ transit and eclipse model, as well as a second-order sinusoidal phase-variation model. The complete astrophysical model had the form

1 $$A(t)={F}_{* }(t)+{F}_{{{{\rm{day}}}}}E(t){{\varPsi }}(\phi ),$$

where *t* is the time, F_*_ is the received stellar flux (and includes the starry transit model), *F*_day_ is the planetary flux at mid-eclipse, *E*(*t*) is the starry eclipse model (neglecting eclipse mapping signals for the purposes of this paper), and *Ψ*(*ϕ*) is the phase-variation model of the form

2 $$\begin{array}{ll}{{\varPsi }}(\phi )=1+\,{{{\rm{AmpCos}}}}1\times (\cos (\phi )-1)+{{{\rm{AmpSin}}}}1\times \sin (\phi )\\\qquad\qquad\,+\,{{{\rm{AmpCos}}}}2\times (\cos (2\phi )-1)+{{{\rm{AmpSin}}}}2\times \sin (2\phi ),\end{array}$$

where *ϕ* is the orbital phase in radians with respect to eclipse, and AmpCos1, AmpSin1, AmpCos2 and AmpSin2 are all fitted coefficients. The second-order phase-variation terms allow for thermal variations across the face of the planet that are more gradual or steep than a simple first-order sinusoid would allow. We numerically computed dayside, morning, nightside and evening spectra using the above *Ψ*(*ϕ*) function at *ϕ* = 0, π/2, π and 3π/2, respectively. To allow the starry eclipse function to account for light travel time, we used a stellar radius (R_*_) of 0.667 *R*_⊙_ (ref. ^55^) to convert the fitted *a*/R_*_ (the scaled semi-major axis) to physical units. For our transit model, we used a reparameterized version of the quadratic limb-darkening model^56^ with coefficients *u*_1_ and *u*_2_ uniformly constrained between 0 and 1, and used a minimally informative prior on the planet-to-star radius ratio (*R*_p_/R_*_).

Our systematics model consisted of a single exponential ramp in time to account for the idle-recovery drift documented for MIRI/LRS time-series observations^29^, a linear trend in time, and a linear trend with the spatial position and PSF width. The full systematics model can be written as

3 $$S(t,y,{s}_{y})=L({t}_{l})\times R({t}_{r})\times Y(\,y)\times SY({s}_{y}),$$

The linear trend in time is modelled as

4 $$L({t}_{{\mathrm{l}}})={c}_{0}+{c}_{1}{t}_{{\mathrm{l}}},$$

where *t*_l_ is the time with respect to the mid-point of the observations and where *c*_0_ and *c*_1_ are coefficients. The exponential ramp is modelled as

5 $$R({t}_{{\mathrm{r}}})=1+{r}_{0}{{\mathrm{e}}}^{{r}_{1}{t}_{{\mathrm{r}}}}$$

where *t*_r_ is the time with respect to the first integration and where *r*_0_ and *r*_1_ are coefficients. The linear trends as a function of spatial position, *y*, are PSF width *s*_*y*_ are modelled as

6 $$Y(\,y)=1+f\,y$$

and

7 $$SY({s}_{y})=1+g{s}_{y},$$

where *f* and *g* are coefficients. The linear trends as a function of spatial position and PSF width are with respect to the mean-subtracted spatial position and PSF width. Finally, we also fitted a multiplier (scatter_mult_) to the estimated Poisson noise level for each integration to allow us to account for any noise above the photon limit as well as an incorrect value for the gain applied in stage 3.

With an initial fit to the broadband light curve (5–10.5 μm), we assumed a zero eccentricity and placed a Gaussian prior on the planet’s orbital parameters (period, *P*; linear ephemeris, *t*_0_; inclination, *i*; and scaled semi-major axis, *a*/R_*_) based on previously published values for the planet^30^. For the fits to the spectroscopic phase curves, we then fixed these orbital parameters to the estimated best fit from the broadband light curve fit to avoid variations in these wavelength-independent values causing spurious features in the final spectra. We fitted the observations using the No U-Turns Sampler (NUTS) from PyMC3^57^ with 3 chains, each taking 6,000 tuning steps and 6,000 production draws with a target acceptance rate of 0.95. We used the Gelman–Rubin statistic^58^ to ensure the chains had converged. We then used the 16th, 50th and 84th percentiles from the PyMC3 samples to estimate the best-fit values and their uncertainties.

#### Eureka! v2 fitting methods

For the second fit made with Eureka!, we proceeded very similarly to the Eureka! v1 fit. We clipped the light curves using a boxcar filter of 20 integrations wide with a maximum of 20 iterations and a rejection threshold of 4*σ* to reject these outliers. We also modelled the phase curve using a second-order sinusoidal function, but we modelled the transit and eclipse using batman^59^ instead of starry. Like in the Eureka! v1 fit, we modelled instrumental systematics with a linear polynomial model in time (equation (4)), an exponential ramp (equation (5)), a first-order polynomial in *y* position (equation (6)) and a first-order polynomial in PSF width in the *s*_*y*_ direction (equation (7)).

We fitted the data using the emcee sampler^60^ instead of NUTS, with 500 walkers and 1,500 steps. The jump parameters that we used were the same as in the Eureka! v1 fit: *R*_p_/R_*_, *F*_day_, *u*_1_, *u*_2_, AmpCos1, AmpSin1, AmpCos2, AmpSin2, *c*_0_, *c*_1_, *r*_0_, *r*_1_, *f*, *g* and scatter_mult_ (multiplier to the estimated Poisson noise level for each integration like in the Eureka! v1 fit). We used uniform priors on *u*_1_ and *u*_2_ from 0 to 1, uniform priors on AmpCos1, AmpSin1, AmpCos2, AmpSin2 from −1.5 to 1.5 and broad normal priors and all other jump parameters. Convergence, mean values and uncertainties were computed in the same way as for the Eureka! v1 fit.

#### TEATRO fitting methods

To measure the planet’s emission as a function of longitude, we modelled the light curves with a phase-variation model, an eclipse model, a transit model and an instrument systematics model. The phase-curve model, *Ψ*(*t*), consists of two sinusoids: one at the planet’s orbital period, *P*, and one at *P*/2 to account for second-order variations. The eclipse model, *E*(*t*), and transit model, *T*(*t*), are computed with the exoplanet^61^ package that uses the starry package^54^. We save the eclipse depth, *δ*_e_, and normalize *E*(*t*) to a value of 0 during the eclipse and 1 out of the eclipse, which we then call *E*_N_(*t*). We used published transit ephemerides^62^, a null eccentricity and published stellar parameters^63^. The planet-to-star radius ratio, *R*_p_/R_*_, impact parameter, *b*, and mid-transit time, *t*_0_, are obtained from a fit to the broadband light curve. The systematics model, *S*(*t*), is composed of a linear function to account for a downwards trend and an exponential function to account for the initial ramp. The full model is expressed as:

8 $$F(t)=({{\varPsi }}(t)-{{\varPsi }}({t}_{{\mathrm{e}}})+{\delta }_{{\mathrm{e}}})\times {E}_{{\mathrm{N}}}(t)+T(t)+S(t)$$

9 $${{\varPsi }}(t)={a}_{{{\varPsi }}}\sin(2\uppi \,t/P-{b}_{{{\Psi }}})+{c}_{{{\varPsi }}}\cos(4\uppi \,t/P-{d}_{{{\varPsi }}})$$

10 $$S(t)={a}_{S}\,{{\mathrm{e}}}^{-{b}_{S}t}+{c}_{S}\,t+{d}_{S}$$

where *Ψ*(*t*_e_) is the value of *Ψ* at the mid-eclipse time, *t*_e_.

We fit our model to the data using a Markov chain Monte Carlo (MCMC) procedure based on the PyMC3 package^57^ and gradient-based inference methods as implemented in the exoplanet package^61^. We set normal priors for *t*_0_, *R*_p_/R_*_, the stellar density (ρ_*_), *a*_*Ψ*_, *b*_*Ψ*_, *c*_*S*_ and *d*_*S*_ with mean values obtained from an initial nonlinear least-squares fit, a normal prior for *a*_*S*_ with a zero mean, uniform priors for the surface brightness ratio between the planet’s dayside and the star (*s*), *b*, *c*_*Ψ*_ and *d*_*Ψ*_, uninformative priors for the quadratic limb-darkening parameters^56^, and allowed for wide search ranges. We ran two MCMC chains with 5,000 tuning steps and 100,000 posterior samples. Convergence was obtained for all parameters (except in one case where *a*_*S*_ was negligible and *b*_*S*_ was unconstrained). We merged the posterior distributions of both chains and used their median and standard deviation to infer final values and uncertainties for the parameters. We also verified that the values obtained from each chain were consistent.

For the spectroscopic light-curve fits, we fixed all physical parameters to those obtained from the broadband light-curve fit except the surface brightness ratio, *s*, that sets the eclipse depth, we masked the transit part of the light curve, and used a similar procedure. After the fits, we calculated the eclipse depth, *δ*_e_, as *s* × (*R*_p_/R_*_)^2^, and computed *Ψ*(*t*) for the final parameters, *Ψ*_f_(*t*). The planetary flux is *Ψ*_f_(*t*) − *Ψ*_f_(*t*_e_) + *δ*_e_. We computed the uncertainty on the eclipse depth in two different ways: from the standard deviation of the posterior distribution of *s* × (*R*_p_/R_*_)^2^, and from the standard deviation of the in-eclipse points divided by $\sqrt{{N}_{{\mathrm{e}}}}$, where *N*_e_ is the number of in-eclipse points, and took the maximum of the two. To estimate the uncertainty on the planet’s flux, we computed the 1*σ* interval of *Ψ*(*t*) based on the posterior distributions of its parameters, computed the 1*σ* uncertainty of *d*_*S*_, and added them in quadrature to the uncertainty on the eclipse depth to obtain more conservative uncertainties.

The spectra presented in this paper and used in the combined spectra are based on system parameters that were derived from a broadband light curve obtained in the 5–12 μm range, a transit fit in which the stellar mass and radius were fixed, a simpler additive model in which the phase curve was not turned off during the eclipse, and an MCMC run that consisted in two chains of 10,000 tuning steps and 10,000 posterior draws. Updated spectra based on system parameters derived from the broadband light curve obtained in the 5–10.5 μm range, a transit fit that has the stellar density as a free parameter, the light-curve model shown in equation (8), and two MCMC chains of 5,000 tuning steps and 100,000 posterior draws are consistent within 1*σ* at every point with those shown here. As we average four reductions and inflate the uncertainties during the retrievals, the impact of these updates on our results are expected to be marginal.

#### SPARTA fitting methods

We use emcee^60^ to fit a broadband light curve with the transit time, eclipse time, eclipse depth, four phase-curve parameters (*C*_1_ and *D*_1_ for the first-order, and *C*_2_ and *D*_2_ for the second-order sinusoids), transit depth, *a*/R_*_, *b*, flux normalization, error-inflation factor, instrumental ramp amplitude (*A*) and 1/timescale (*τ*), linear slope in time (*m*) with respect to the mean of the integration times $(\overline{t})$, and linear slope with trace *y* position (*c*_*y*_) as free parameters. The best-fit transit and eclipse times, *a*/R_*_ and *b* are fixed for the spectroscopic light curves.

For the spectroscopic fits, we then use emcee to fit the free parameters: the eclipse depth, four phase-curve parameters, error-inflation factor, flux normalization, instrumental ramp amplitude and 1/timescale, linear slope with time, and linear slope with trace *y* position. All parameters are given uniform priors. 1/timescale is given a prior of 5–100 d^−1^, but the other priors are unconstraining. In summary, the instrumental model is:

11 $$S={F}_{*}\left(1+A\exp (-t/\tau )+{c}_{y}y+m(t-\overline{t})\right),$$

while the planetary flux model is:

12 $${F}_{{\mathrm{p}}}=E+{C}_{1}(\cos (\omega t)-1)+{D}_{1}\sin (\omega t)+{C}_{2}(\cos (2\omega t)-1)+{D}_{2}\sin (2\omega t),$$

where *E* is the eclipse depth and *ω* = 2π/*P* is the planet’s orbital angular frequency. Note that the phase variations were set to be zero during eclipse.

#### Combining independent spectra

Comparing the phase-resolved spectra from each reduction (Supplementary Fig. 2), we see that for wavelengths below 10.5 μm, the spectra are typically consistent, while larger differences arise in the >10.5 μm region affected by the shadowed region effect. For our final, fiducial spectrum, we decided to use the median spectrum and inflated our uncertainties to account for disagreements between different reductions. The median phase-resolved spectra were computed by taking the median *F*_p_/F_*_ per wavelength. The uncertainties were computed by taking the median uncertainty per wavelength, and then adding in quadrature the RMS between the individual reductions and the median spectrum; this minimally affects the uncertainties where there is minimal disagreement and appreciably increases the uncertainties where the larger disagreements arise.

Each reduction also computed a transmission spectrum (Supplementary Fig. 2), which appears quite flat (within uncertainties) with minimal differences between reductions. WASP-43b is not an excellent target for transmission spectroscopy, however, and these transmission spectra are not expected to be overly constraining.

### Atmospheric forward models

GCMs were used to simulate atmospheric conditions, from which synthetic phase curves and emission spectra were forward modelled and compared with the observations. The GCMs used in this study are listed in Supplementary Table 1, and details of each simulation are provided in Extended Data Table 1 and the following sections.

#### Generic PCM

The Generic Planetary Climate Model (Generic PCM) is a three-dimensional global climate model designed for modelling the atmosphere of exoplanets and for palaeoclimatic studies. The model has been used for the study of planetary atmospheres of the Solar System^64–66^, terrestrial exoplanets^67^, mini-Neptunes^68^ and hot Jupiters^69^. The dynamical core solves the primitive equations of meteorology on a Arakawa C grid. The horizontal resolution is 64 × 48 (that is, 5.625 × 3.75°) with 40 vertical levels, equally spaced in logarithmic scale between 10 Pa and 800 bars. Along with the various parameterizations of physical processes described in refs. ^64–68^, the Generic PCM treats clouds as radiatively active tracers of fixed radii.

The model is initialized using temperature profiles from the radiative–convective one-dimensional model Exo-REM^70^. The radiative data are computed offline using the out-of-equilibrium chemical profiles of the Exo-REM run. We use 27 frequency bins in the stellar channel (0.261–10.4 μm) and 26 in the planetary channel (0.625–324 μm), all bins including 16 *k*-coefficients. We start the model from a rest state (no winds), with a horizontally homogeneous temperature profile. Models are integrated for 2,000 days, which is long enough to complete the spin-up phase of the simulation above the photosphere. We do not include Rayleigh drag in our models. The simulations are performed including clouds of Mg_2_SiO_4_, with varying cloud radii (0.1, 0.5, 1, 3 and 5 μm). We also computed cloudless and Mg_2_SiO_4_ models with a 10× solar metallicity and the same radii for the cloud particles. Regardless of the composition and size of the clouds, our model clearly indicates that there is no cloud formation on the dayside. Asymmetric limbs are a natural result of our model, with the eastern terminator being warmer while the western limb is cloudier and cooler. Spectral phase curves were produced using the Pytmosph3R code^71^.

#### SPARC/MITgcm with radiative transfer post-processing by gCMCRT

SPARC/MITgcm couples a state-of-the-art non-grey, radiative-transfer code with the MITgcm^33^. The MITgcm solves the primitive equations of dynamical meteorology on a cubed-sphere grid^72^. It is coupled to the non-grey radiative-transfer scheme based on the plane-parallel radiative-transfer code of ref. ^73^. The stellar irradiation incident on WASP-43b is computed with a PHOENIX model^74–76^. We use previously published opacities^77^, including more recent updates^78,79^, and the molecular abundances are calculated assuming local chemical equilibrium^80^. In the GCM simulations, the radiative-transfer calculations are performed on 11 frequency bins ranging from 0.26 μm to 300 μm, with 8 *k*-coefficients per bin statistically representing the complex line-by-line opacities^3^. The strong visible absorbers TiO and VO are excluded in our *k* tables similar to our previous GCMs of WASP-43b^3,23^ that best match the observed dayside emission spectrum and photometry.

Clouds in the GCM are modelled as tracers that are advected by the flow^81^ and can settle under gravity. Their formation and evaporation are subjected to chemical equilibrium predictions, that is, the condensation curves of various minerals described in ref. ^80^. The conversion between the condensable ‘vapour’ and clouds is treated as a simple linear relaxation over a short relaxation timescale of 100 s. The scattering and absorption of the spatial- and time-dependent clouds are included in both the thermal and visible wavelengths of the radiative transfer. A similar dynamics–cloud–radiative coupling has been developed in our previous GCMs with simplified radiative transfer and has been used to study the atmospheric dynamics of brown dwarfs^9,82^ and ultrahot Jupiters^83^. Clouds are assumed to follow a log-normal size distribution^84^, which is described by the reference radius *r*_0_ and a non-dimensional deviation *σ*: $n(r)=\frac{{{{\mathcal{N}}}}}{\sqrt{2\uppi }\sigma r}\exp \left(-\frac{{[\ln (r/{r}_{0})]}^{2}}{2{\sigma }^{2}}\right)$, where *n*(*r*) is the number density per radius bin of *r* and ${{{\mathcal{N}}}}$ is the total number density. *σ* and *r*_0_ are free parameters and the local ${{{\mathcal{N}}}}$ is obtained from the local mass mixing ratio of clouds. The size distribution is held fixed throughout the model and is the same for all types of cloud.

Our GCMs do not explicitly impose a uniform radiative heat flux at the bottom boundary but rather relax the temperature of the lowest model layer (that is, the highest pressure layer) to a certain value over a short timescale of 100 s. This assumes that the deep GCM layer reaches the convective zone and the temperature there is set by the interior convection that ties to the interior structure of the planet. This lowest-layer temperature is in principle informed by internal structure models of WASP-43b, which are run by MESA hot Jupiter evolution modules^12^ to match the present radius of WASP-43b. In most models, this lowest-layer temperature is about 2,509 K at about 510 bars. The horizontal resolution of our GCMs is typically C48, equivalent to about 1.88° per grid cell. The vertical domain is from 2 × 10^−4^ bar at the top to 700 bars at the bottom and is discretized to 53 vertical layers. We typically run the simulation for over 1,200 days and average all physical quantities over the last 100 days of the simulations.

All our GCMs assume a solar composition. We performed a baseline cloudless model and one case with only MnS and Na_2_S clouds with *r*_0_ = 3 μm, and then a few cases with MnS, Na_2_S and MgSiO_3_ clouds with *r*_0_ = 1, 1.5, 2 and 3 μm. The *σ* is held fixed at 0.5 in all our cloudy GCMs.

We post-process our GCM simulations with the state-of-the-art gCMCRT code, which is a publicly available hybrid Monte Carlo radiative transfer (MCRT) and ray-tracing radiative-transfer code. The model is described in detail in ref. ^85^ and has been applied to a range of exoplanet atmospheres^83,86^. gCMCRT can natively compute albedo, transmission and emission spectra at both low and high spectral resolution. gCMCRT uses custom *k* tables, which take cross-section data from both HELIOS-K^87^ and EXOPLINES^88^. Here, we apply gCMCRT to compute low-resolution emission spectra and phase curves at *R* ≈ 300 from our GCM simulations. We use the three-dimensional temperature and condensate cloud tracer mixing ratio from the time-averaged end-state of each case. We assume the same cloud particle size distribution as our GCMs.

#### expeRT/MITgcm

The GCM expeRT/MITgcm uses the same dynamical core as SPARC/MITgcm and solves the hydrostatic primitive equations on a C32 cubed-sphere grid^72^. It resolves the atmosphere above 100 bar on 41 log-spaced cells between 1 × 10^−5^ bar and 100 bar. Below 100 bar, 6 linearly spaced grid cells between 100 bar and 700 bar are added. The model expeRT/MITgcm thus resolves deep dynamics in non-inflated hot Jupiters like WASP-43b^16,89^.

The GCM is coupled to a non-grey radiative-transfer scheme based on petitRADTRANS^90^. Fluxes are recalculated every fourth dynamical time step. Stellar irradiation is described by the spectral fluxes from the PHOENIX model atmosphere suite^74–76^. The GCM operates on a precalculated grid of correlated *k*-binned opacities. Opacities from the ExoMol database^91^ are precalculated offline on a grid of 1,000 logarithmically spaced temperature points between 100 K and 4,000 K for every vertical layer. We further include the same species as shown in ref. ^89^ except TiO and VO to avoid the formation of a temperature inversion in the upper atmosphere. These are: H_2_O (ref. ^92^), CH_4_ (ref. ^93^), CO_2_ (ref. ^94^), NH_3_ (ref. ^95^), CO (ref. ^96^), H_2_S (ref. ^97^), HCN (ref. ^98^), PH_3_ (ref. ^99^), FeH (ref. ^100^), Na (refs. ^74,101^) and K (refs. ^74,101^). For Rayleigh scattering, the opacities are H_2_ (ref. ^102^) and He (ref. ^103^), and we add the following collision-induced absorption (CIA) opacities: H_2_–H_2_ (ref. ^104^) and H_2_–He (ref. ^104^). We use for radiative-transfer calculations in the GCM the same wavelength resolution as SPARC/MITgcm (S1), but incorporate 16 instead of 8 *k*-coefficients. Two cloud-free WASP-43b GCM simulations were performed, one with solar and one with 10× solar element abundances. Each simulation ran for 1,500 days to ensure that the deep wind jet has fully developed. The GCM results used in this paper were time averaged over the last 100 simulation days.

Spectra and phase curves are produced from our GCM results in post-processing with petitRADTRANS^90^ and prt_phasecurve^89^ using a spectral resolution of *R* = 100 for both the phase curve and the spectra.

#### RM-GCM

Originally adapted from the GCM of ref. ^105^ by refs. ^106–108^, the RM-GCM model has been applied to numerous investigations of hot Jupiters and mini-Neptunes^35,109–111^. The GCM’s dynamical core solves the primitive equations of meteorology using a spectral representation of the domain, and it is coupled to a two-stream, double-grey radiative-transfer scheme based on ref. ^112^. Recent updates have added aerosol scattering^35^ with radiative feedback^8,36^. Following ref. ^8^, aerosols are representative of condensate clouds and are treated as purely temperature-dependent sources of opacity, with constant mixing ratios set by the assumed solar elemental abundances. The optical thicknesses of the clouds are determined by converting the relative molecular abundances (or partial pressures) of each species into particles with prescribed densities and radii^8^. The model includes up to 13 different cloud species of various condensation temperatures, abundances and scattering properties. Places where clouds overlap have mixed properties, weighted by the optical thickness of each species.

Simulations from this GCM included a clear atmosphere and two sets of cloudy simulations. Following ref. ^8^, one set of cases included 13 different species: KCl, ZnS, Na_2_S, MnS, Cr_2_O_3_, SiO_2_, Mg_2_SiO_4_, VO, Ni, Fe, Ca_2_SiO_4_, CaTiO_2_ and Al_2_O_3_; the other set omitted ZnS, Na_2_S, MnS, Fe and Ni, based on considerations of nucleation efficiency^113^. For both cloud composition scenarios, the models explored the observational consequences of variations in the cloud deck’s vertical thickness through a series of simulations with clouds tops truncated over a range of heights at 5-layer intervals (roughly a scale height), ranging from 5 to 45 layers of the 50-layer model. This effectively mimics a range of vertical mixing strengths. From the complete set published in ref. ^26^, we selected a subset, with clouds of maximum vertical extents between two and nine scale heights from each of the two cloud composition scenarios.

Simulations were initialized with clear skies, no winds and no horizontal temperature gradients. We ran the simulations for over 3,500 planetary orbits, assuming tidal synchronization. Resulting temperature, wind and cloud fields of the GCM were then post-processed^114,115^ to yield corresponding emission phase curves.

#### THOR

THOR^116,117^ is an open-source GCM developed to study the atmospheres and climates of exoplanets, free from Earth- or Solar System-centric tunings. The core that solves the fluid flow equations, the dynamical core, solves the non-hydrostatic compressible Euler equations on an icosahedral grid^116,118^. THOR has been validated and used to simulate the atmosphere of Earth^116,119^, Solar System planets^120,121^ and exoplanets^116,117,122^.

For this work, THOR used the same configuration as with previously published simulations to study the atmospheric temperature structure, cloud cover and chemistry of WASP-43b^4,38,123^. Two simulations were conducted, one with a clear atmosphere and another with a cloud structure on the nightside of the planet. To represent the radiative processes, THOR uses a simple two-band formulation calibrated to reproduce the results from more complex non-grey models on WASP-43b^3,124^. A simple cloud distribution on the nightside of the planet and optical cloud properties are parameterized^4^ and adapted to reproduce previous HST^21^ and Spitzer^4,22^ observations. These simulations on WASP-43b with THOR have also been used to test the performance of future Ariel phase-curve observations^125^.

Both simulations, with clear and cloudy atmospheres, started with isothermal atmospheres (1,440 K, equilibrium temperature) and integrated for roughly 9,400 planetary orbits (assuming a tidally locked configuration) until a statistically steady state of the deep atmosphere thermal structure was reached. The long integration avoids biasing the results towards the set initial conditions^120^.

The multiwavelength spectra are obtained from post-processing the three-dimensional simulations with a multiwavelength radiative-transfer model^126^. The disk-averaged planet spectrum is calculated at each orbital phase by projecting the outgoing intensity for each geographical location of the observed hemisphere. The spectra include cross-sections of the main absorbers in the infrared, drawn from the ExoMOL (H_2_O (ref. ^92^), CH_4_ (ref. ^127^), NH_3_ (ref. ^128^), HCN (ref. ^129^) and H_2_S (ref. ^97^)), HITEMP^130^ (CO_2_ and CO) and HITRAN^131^ (C_2_H_2_) databases. The Na and K resonance lines^132^ are also added, as were H_2_–H_2_ and H_2_–He CIA^104^. The atmospheric bulk composition was assumed to have solar abundance (consistent with HST/WFC3 spectrum observations), and each chemical species concentration was calculated with the FastChem model^133^. The PHOENIX models^74–76^ were used for the WASP-43 star spectrum.

### Atmospheric retrieval models

We perform atmospheric retrievals on the phase-resolved emission spectra using six different retrieval frameworks, each described in turn below. The chemical constraints from these analyses are summarized in Extended Data Tables 2 and 3, and the spectral fits obtained are shown in Extended Data Fig. 3. Across the six retrieval analyses, we use an error-inflation parameter to account for the effects of unknown data and/or model uncertainties. This free parameter is wavelength independent and multiplies the 1*σ* error bars in the calculation of the likelihood function in the Bayesian sampling algorithm.

#### HyDRA retrieval framework

The HyDRA atmospheric retrieval framework^134^ consists of a parametric atmospheric forward model coupled to PyMultiNest^135,136^, a nested sampling Bayesian parameter estimation algorithm^137^. HyDRA has been applied to hydrogen-rich atmospheres^138,139^, and further adapted for secondary atmospheres^140^ and high-resolution spectroscopy in both one and two dimensions^141,142^. The input parameters for the atmospheric forward model include constant-with-depth abundances for each of the chemical species considered, six temperature profile parameters corresponding to the temperature profile model of ref. ^143^, and a constant-with-wavelength multiplicative error-inflation parameter to account for model uncertainties. We additionally include a dilution parameter, *A*_HS_, for the dayside, morning and evening hemispheres, which multiplies the emission spectrum by a constant factor <1 and accounts for temperature inhomogeneities in each hemisphere^144^.

We consider opacity contributions from the chemical species that are expected to be present in hot Jupiter atmospheres and that have opacity in the MIRI LRS wavelength range: H_2_O (ref. ^130^), CH_4_ (refs. ^127,145^), NH_3_ (ref. ^128^), HCN (refs. ^98,129^), CO (ref. ^130^), CO_2_ (ref. ^130^), C_2_H_2_ (refs. ^131,146^), SO_2_ (ref. ^147^), H_2_S (refs. ^97,148^) and CIA due to H_2_–H_2_ and H_2_–He (ref. ^104^). The line-by-line absorption cross-sections for these species are calculated following the methods described in ref. ^134^, using data from each of the references listed. We further explore retrievals with a simple silicate cloud model, which includes the modal particle size, cloud particle abundance, cloud base pressure and a pressure exponent for the drop-off of cloud particle number density with decreasing pressure. The opacity structure of the cloud is calculated using the absorption cross-sections of ref. ^149^.

Given the input chemical abundances, temperature profile and cloud parameters, the forward model calculates line-by-line radiative transfer to produce the thermal emission spectrum at a resolution of *R* ≈ 15,000. The spectrum is then convolved to a resolution of 100, binned to the same wavelength bins as the observations and compared with the observed spectrum to calculate the likelihood of the model instance. The nested sampling algorithm explores the parameter space using 2,000 live points, and further calculates the Bayesian evidence of the retrieval model, which can be used to compare different models^52^. In particular, we calculate the detection significance of a particular chemical species by comparing retrievals that include/exclude that species, fixing the value of the error-inflation parameter to be the median retrieved value found with the full retrieval model.

Across the four phases, the only chemical species detected with statistical significance (≳3*σ*) is H_2_O. The retrieved H_2_O abundances are in the range ~30–10^4^ ppm (1*σ* uncertainties), with detection significances varying between ~3*σ* and ~4*σ* (Extended Data Table 2). We do not detect CH_4_ at any phase, and place an upper limit of 16 ppm on the nightside CH_4_ abundance, potentially indicating disequilibrium chemistry processes as described in the main text. We do not detect NH_3_ at any phase either, consistent with the very low NH_3_ abundances predicted by both chemical equilibrium and disequilibrium models^23^. The retrievals do not favour cloudy models over clear models with statistical significance, with extremely weak preferences of <1*σ* at all phases. In addition, the posterior probability distributions for the cloud parameters are unconstrained. Extended Data Fig. 5 shows the pressure ranges of the atmospheric model probed by the observations.

#### PyratBay retrieval framework

PyratBay is an open-source framework that enables atmospheric modelling, spectral synthesis, and atmospheric retrievals of exoplanet observations^150^. The atmospheric model consists of parametric temperature, composition and altitude profiles as a function of pressure, for which emission and transmission spectra can be generated. The radiative-transfer module considers opacity from alkali lines^151^, Rayleigh scattering^152,153^, Exomol and HITEMP molecular line lists^130,154^ pre-processed with the REPACK package^155^ to extract the dominant line transitions, CIA^156^ and cloud opacities. The PyratBay retrieval framework has the ability to stagger model complexity and explore a hierarchy of different model assumptions. Temperature models range from an isothermal profile to physically motivated parameterized models^143,157^. Composition profiles range from the simpler constant-with-altitude ‘free abundance’ to the more complex ‘chemically consistent’ retrievals, the latter accomplished via the TEA code^158^; while cloud condensate prescriptions range from the classic ‘power law + grey’ to a ‘single-particle-size’ haze profile, a partial-coverage factor ‘patchy clouds’^159^, and the complex parameterized Mie-scattering thermal stability cloud (TSC) model (J.B. et al., manuscript in preparation). The TSC cloud prescription, initially inspired by refs. ^84,160^, has additional flexibility in the location of the cloud base and was further improved for this analysis (see below). The formulation utilizes a parameterized cloud shape, effective particle size and gas number density below the cloud deck, while the atmospheric mixing and settling are wrapped up inside the cloud extent and the condensate mole fraction as free parameters. This cloud model was applied to WASP-43b JWST/MIRI phase-curve simulations^23^, generated during the JWST preparatory phase, in anticipation of the actual WASP-43b JWST/MIRI observations. We showed that the TSC model has the ability to distinguish between MgSiO_3_ and MnS clouds on the nightside of the planet.

For this analysis, we conducted a detailed investigation using various model assumptions. We started by exploring simple temperature prescriptions and gradually moved towards more complex ones. Initially, we considered opacity contributions from all chemical species expected to be observed in the MIRI wavelength range (H_2_O, CH_4_, NH_3_, HCN, CO, CO_2_, C_2_H_2_, SO_2_, H_2_S), but eventually focused on only those that are fit by the data. We also implemented the dilution parameter^144^ and an error-inflation factor, which account for some additional model and data uncertainties. The constraints on H_2_O (together with the detection significance^161^) and the upper limit for CH_4_ for all phases are given in Extended Data Table 2. The abundances of these species across all phases were largely model independent. However, the tentative constraints on NH_3_, which we saw in multiple phases, were strongly model dependent, and were completely erased with the inclusion of the dilution parameter and the error inflation, thus we do not report them here. WASP-43b emission spectra were computed at a resolution of *R* ≈ 15,000 utilizing opacity sampling of high-resolution pre-computed cross-sections (*R* ≈ 10^6^) of considered species. Furthermore, we thoroughly examined the possibility of detecting clouds in each of the four-quadrant phases, with a special emphasis on the nightside of the planet. To do this, we employed the TSC model, as in our previous analysis^23^, and explored a range of cloud species, MgSiO_3_, MnS, ZnS and KCl, that would condense under the temperature regimes expected for WASP-43b^162^ (Extended Data Fig. 6, left). We also introduced the effective standard deviation of the log-normal distribution^84^ as a free parameter (*σ*_log_), allowing for even more flexibility in our cloud model (Extended Data Fig. 6, right, last subpanel). To thoroughly explore the parameter space, we used two Bayesian samplers, the differential-evolution MCMC algorithm^163^, implemented following ref. ^164^, and the nested sampling algorithm, implemented through PyMultiNest^135,136^, utilizing 15 million models and 2,000 live points, respectively. Our investigation did not provide constraints on any of the cloud parameters for any of the explored cloud condensates at any of the planetary phases, indicating the absence of detectable spectral features from clouds in the observations (Extended Data Fig. 6, right).

#### NEMESIS retrieval framework

NEMESIS^165,166^ is a free retrieval framework that uses a fast correlated-*k*^167^ forward model, combined with either an optimal estimation or nested sampling retrieval algorithm. It has been used to perform retrievals on spectra of numerous planetary targets, both inside and outside the Solar System^168,169^. In this work, we use the PyMultiNest sampler^136^ with 500 live points. The retrieval model presented includes four spectrally active gases, H_2_O (ref. ^92^), CO (ref. ^96^), CH_4_ (ref. ^93^) and NH_3_ (ref. ^95^), with *k* tables calculated as in ref. ^91^; we did not include CO_2_ or H_2_S after initial tests indicated these were not required to fit the spectrum. All gases are assumed to be well mixed in altitude. CIA from H_2_ and He is taken from refs. ^156,170^. The spectrum is calculated at the resolution of the observation, using optimized channel integrated *k* tables generated from original *k* tables with a resolving power *R* = 1,000. The temperature profile is modelled as a three-parameter Guillot profile, after ref. ^157^, with free parameters *κ*, *γ* and *β* (*α* is fixed to be zero). We include a well-mixed, spectrally grey cloud with a scalable total optical depth with a cloud top at 12.5 mbar. The other retrieved parameters are a hotspot dilution factor for phases 0.25, 0.5 and 0.75, following ref. ^144^, and an error-inflation term.

To calculate the detection significance for H_2_O, we run the retrieval with and without H_2_O, with all other aspects of the run identical. We then take the difference of the PyMultiNest global log-evidence values for the two scenarios, and convert from log(Bayesian evidence) to sigma following ref. ^52^. The 99% upper limit for CH_4_ is calculated from the equally weighted posterior distribution. We also attempt to retrieve CO and NH_3_ abundances. CO is generally poorly constrained, and NH_3_ is unconstrained for phases 0 and 0.75; for log(NH_3_), we recover a 99% upper limit of −2.2 at phase 0.25 and −3.9 at phase 0.5. The cloud opacity is also generally unconstrained, with the total optical depth able to span several orders of magnitude. We stress that this model is very crude as it has only one variable cloud parameter, and further exploration of suitable cloud models for mid-infrared phase curves is warranted in future work.

#### SCARLET retrieval framework

We perform atmospheric retrievals on the four phase-resolved spectra using the SCARLET framework^160,171^. The planetary disk-integrated thermal emission, *F*_p_, is modelled for a given set of atomic/molecular abundances, temperature–pressure profile and cloud properties. We compare our model spectra with the observations by normalizing the thermal emission *F*_p_ using a PHOENIX^74–76^ stellar model spectrum with effective temperature *T*_eff_ = 4,300 K and surface gravity log *g* = 4.50. The model spectra are computed at a resolving power of *R* = 15,625, convolved to the resolving power of MIRI/LRS and then binned to the 11 spectral bins (<10.5 μm) considered in the analysis, assuming the throughput to be uniform over a single bin.

The atmospheric analysis is performed considering thermochemical equilibrium, where the metallicity [M/H] (${{{\mathcal{U}}}}[-3,3]$) and carbon-to-oxygen ratio (${{{\mathcal{U}}}}[0,3]$) are free parameters that dictate the overall atmospheric composition. We use a free parameterization of the temperature–pressure profile^172^ by fitting for *N* = 4 temperature points (${{{\mathcal{U}}}}[100,4400]\,{\mathrm{K}}$) with a constant spacing in log-pressure. The temperature–pressure profile is interpolated to the 50 layers (*P* = 10^2^–10^−6^ bar) considered in the model using a spline function to produce a smooth profile. We use a grid of chemical equilibrium abundances produced with FastChem2^173^ to interpolate the abundance of species as a function of temperature and pressure for given values of [M/H] and C/O. The species considered in the equilibrium chemistry are H, H^−^ (refs. ^174,175^), H_2_, He, H_2_O (ref. ^92^), OH (ref. ^130^), CH_4_ (ref. ^127^), C_2_H_2_ (ref. ^176^), CO (ref. ^130^), CO_2_ (ref. ^130^), NH_3_ (ref. ^95^), HCN (ref. ^98^), PH_3_ (ref. ^99^), TiO (ref. ^177^) and VO (ref. ^178^). All opacities for these species are considered when computing the thermal emission. We account for potential spatial atmospheric inhomogeneities in the planetary disk that are observed at a given phase by including an area fraction parameter *A*_HS_ (${{{\mathcal{U}}}}[0,1]$), which is meant to represent the possibility of a fraction of the disk contributing to most of the observed thermal emission^144^. This parameter is considered for all phases with the exception of the nightside, which is expected to be relatively uniform. Finally, we fit for an error-inflation parameter *k*_*σ*_ (${{{\mathcal{U}}}}[0.1,10]$) to account for potential model and data uncertainty, which results in a total of 8 (7 for the nightside) free parameters. We consider 8 walkers per free parameter for the retrievals which are run for 30,000 steps. The first 18,000 steps are discarded when producing the posterior distributions of the free parameters.

#### PLATON retrieval framework

PLATON^179^, Planetary Atmosphere Tool for Observer Noobs, is a Bayesian retrieval tool that assumes equilibrium chemistry. We adopt the temperature–pressure profile parameterization of ref. ^180^, and use the dynesty nested sampler^49^ to retrieve the following free parameters: stellar radius; stellar temperature; the log metallicity, log(*Z*); C/O; 5 temperature–pressure parameters (log(*κ*_th_), log(*γ*), log(*γ*_2_), *α*, *β*); and an error multiplier. The stellar radius and temperature are given Gaussian priors with means and standard deviations set by the measurements in ref. ^55^: 4,400 ± 200 K and 0.667 ± 0.011 *R*_⊙_, respectively. The combination of the two have a similar effect to the dilution parameter of other retrieval codes, which multiplies the emission spectrum by a constant. For phase 0.0, we obtain a significantly better fit when methane opacity is set to zero (thus removing all spectral features from methane). We therefore adopt this as the fiducial model, whereas for other phases, we do not zero out any opacities.

For all retrievals, we use nested sampling with 1,000 live points. The opacities (computed at *R* = 10,000) and the line lists used to compute them are listed in ref. ^179^. We include all 31 species in retrieval, notably including H_2_O, CO, CO_2_, CH_4_ (except on the nightside), H_2_S and NH_3_.

#### ARCiS retrieval framework

ARCiS (Artful modelling code for exoplanet science) is an atmospheric modelling and Bayesian retrieval code^181,182^ that utilizes the MULTINEST^135^ Monte Carlo nested sampling algorithm. The code was used in previous retrievals of the atmosphere of WASP-43b in transmission^183^, using the observations of ref. ^184^, and in phase-resolved emission^185^, using the observations of refs. ^21,22,25,186^. Reference ^183^ found some evidence that AlO improves the fit of the transmission spectra of WASP-43b in the 1.1–1.6 μm region. We therefore include in our models for this work the following set of molecules in our free molecular retrievals: H_2_O (ref. ^92^), CO (ref. ^96^), CO_2_ (ref. ^94^), NH_3_ (ref. ^95^), CH_4_ (ref. ^93^) and AlO (ref. ^187^). The molecular line lists are from the ExoMol^154,188^ or HITEMP^130^ databases as specified, and *k* tables from the ExoMolOP opacity database^91^. CIA for H_2_ and He are taken from refs. ^156,170^. We explore the inclusion of a variety of additional molecules that have available line list data with spectral features in the region of our observations, including HCN (ref. ^98^), SiO (ref. ^189^) and N_2_O (ref. ^130^). We use the Bayes factor, which is the difference between the nested sampling global log-evidence (log *E*) between two models, to assess whether the inclusion of a particular parameter is statistically significant. For this, we run a retrieval with the base set of species only and another with the base set plus the molecule being assessed. The difference in log *E* between the two models is converted to a significance in terms of *σ* using the metric of ref. ^52^. We explore the inclusion of a simple grey, patchy cloud model, which parameterizes cloud top pressure and degree of cloud coverage (from 0 for completely clear to 1 for completely covered). We use 1,000 live points and a sampling efficiency of 0.3 in MULTINEST for all retrievals.

We run retrievals both including and not including a retrieved error-inflation parameter. The error-inflation parameter is implemented as per ref. ^190^ to account for underestimated uncertainties and/or unknown missing forward model parameters. All phases apart from 0.0 retrieved a parameter that increases the observational error bars by two to three times their original values. The pressure–temperature profile parameterization of ref. ^191^ is used in all cases. We find evidence for the inclusion of H_2_O for all four phases, although this evidence goes from strong to weak when error inflation is included for the morning phase (0.75). We find no strong evidence for CH_4_ at any phase, with 95% confidence upper limits on the log of the volume mixing ratio (VMR) of −4.9, −2.9, −3.2 and −2.2 for phases 0.0, 0.25, 0.5 and 0.75, respectively. We find some model-dependent hints of moderate evidence (based on the metric of ref. ^52^) of 4.4*σ* for NH_3_ at phase 0.5 (constrained to $\log{{\mathrm{VMR}}}={-4.5}_{-0.5}^{+0.7}$), 3.1*σ* for CO at phase 0.5 ($\log{{\mathrm{VMR}}}={-1.7}_{-0.7}^{+0.5}$) and 2.6*σ* for CO at phase 0.25 ($\log{{\mathrm{VMR}}}={-4.0}_{-0.4}^{+0.3}$). However, these disappear when the error-inflation parameter is introduced. We are not able to constrain any of the cloud parameters for any phase, and so do not find a statistical reason to include our simple cloud parameterization in the models to better fit the observations.

### Supplementary information

Supplementary Information Supplementary Table 1 and Figs. 1 and 2.

## Figures and Tables

**Extended Data Table 1:**
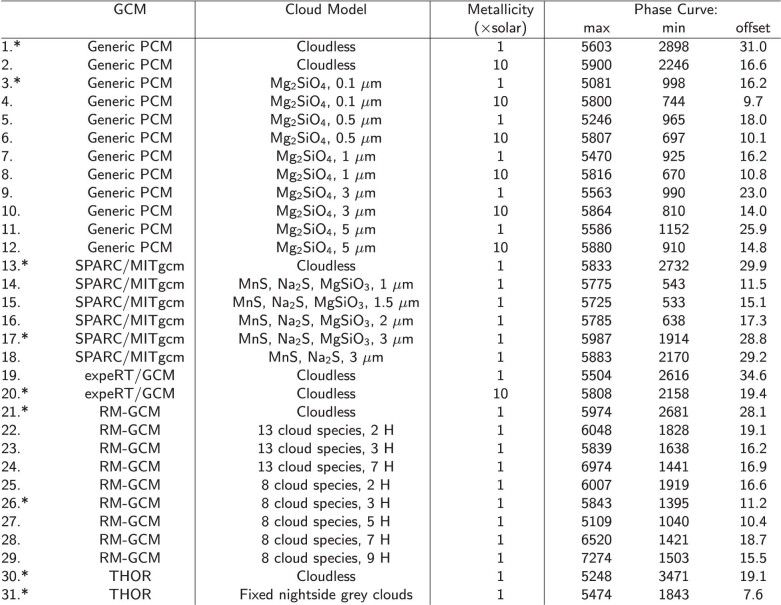
General Circulation Model simulations used in this investigation

For cloud models, the composition of the condensates are provided, along with the prescribed mean radius of the condensed cloud particles. For the RM-GCM cloud models, simulations with eight species of clouds include KCl, Cr_2_O_3_, SiO_2_, Mg_2_SiO_4_, VO, Ca_2_SiO_4_, CaTiO_2_, and Al_2_O_3_, while those with 13 species additionally include ZnS, Na_2_S, MnS, Fe, and Ni; in these cases, particle sizes vary with height, and the prescribed maximum vertical extent of the clouds, expressed in pressure scale heights (H), are also provided. Further details of the models can be found in the text. Phase curve maxima and minima are expressed as the planet-to-star flux ratio in ppm, while offsets are expressed in degrees of longitude east relative to the sub-stellar longitude. Simulations featured in Figure 3 are indicated (*).

**Extended Data Table 2:**
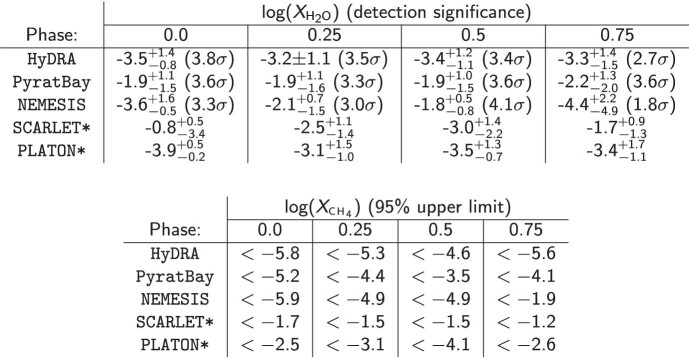
Inferences on H_2_O and CH_4_ abundances and detection significances

The H_2_O volume mixing ratios and CH_4_ upper limits inferred by each retrieval framework at each phase, in log values. H_2_O detection significances are shown in brackets where applicable. Codes marked with * assume equilibrium chemistry and cannot constrain the abundances of molecules individually. For these codes, we report the abundance of water at 100 mbar.

**Extended Data Table 3:**
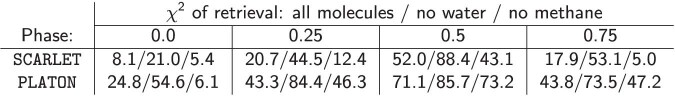
Chi-squared values of equilibrium chemistry retrievals with and without opacity from water and methane

The retrieval setup is identical aside from the inclusion or exclusion of opacity from water or methane. The results presented here do not consider the error-inflation parameter (the retrievals would be free to inflate errors until a *χ*^2^/N_points_ value of 1 is reached). The models were fit to the phase-resolved spectra which each had 11 data points. The PLATON retrieval included 9 parameters. The SCARLET retrieval included 7 parameters for phases 0.25, 0.5, and 0.75, and 6 parameters for phase 0.0.

## Data Availability

The data used in this paper are associated with JWST DD-ERS programme 1366 (principal investigators N.M.B., J.L.B. and K.B.S.; observation 11) and are publicly available from the Mikulski Archive for Space Telescopes (https://mast.stsci.edu). Additional intermediate and final results from this work are archived on Zenodo at 10.5281/zenodo.10525170 (ref. ^192^).

## Code availability

We used the following codes to process, extract, reduce and analyse the data: STScI’s JWST Calibration pipeline^46^, Eureka!^45^, TEATRO, SPARTA^48^, Generic PCM^64–68^, SPARC/MITgcm^9,33,81,82^, expeRT/GCM^16,72,89^, RM-GCM^8,35,106–108^, THOR^4,116,117,124,126,193^, HyDRA^134^, PyratBay^150^, NEMESIS^165,166^, SCARLET^160,171^, PLATON^179^, starry^54^, exoplanet^61^, PyMC3^57^, emcee^60^, dynesty^49^, numpy^194^, astropy^195,196^ and matplotlib^197^.
