# Supplementary material for: Nightside clouds and disequilibrium chemistry on the hot Jupiter WASP-43b
Source: Nat Astron. 2024 Apr 30;8(7):879–98. doi: 10.1038/s41550-024-02230-x (PMC11263128; doi:10.1038/s41550-024-02230-x)
Supplement: Supplementary file 1 — Supplementary Table 1 and Figs. 1 and 2. [file 41550_2024_2230_MOESM1_ESM.pdf]

nature astronomy

Article

https://doi.org/10.1038/s41550-024-02230-x

Nightside clouds and disequilibrium chemistry on the hot Jupiter WASP-43b

In the format provided by the authors and unedited

Supplementary Information Tables

Supplementary Table 1: Summary of the five GCMs used in this study.

GCM Name	Radiative Transfer	Post-processing	References
Generic PCM	non-grey correlated k	Pytmosph3R	[69–73]
SPARC/MITgcm	non-grey correlated k	gCMCRT	[9,33,83,84]
expeRT/GCM	non-grey correlated k	petitRADTRANS	[16,77,91]
RM-GCM	double-grey	unnamed[116,117]	[8,35,108–110]
THOR	double-grey	HELIOS	[4,118,119,126,128,198]

Supplementary Information Figures

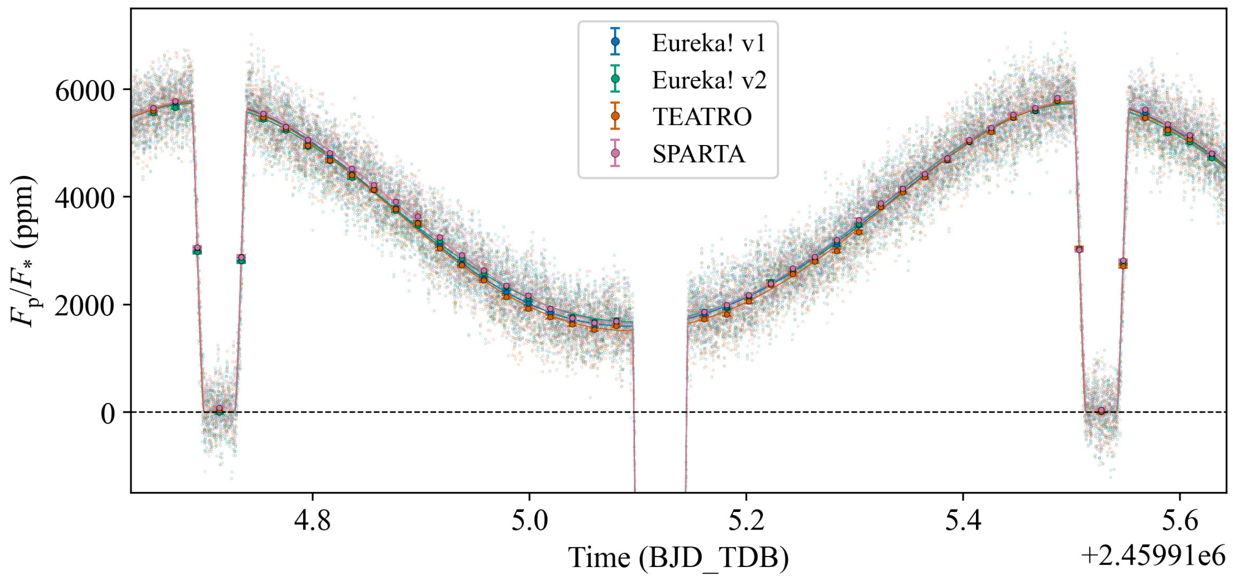

Supplementary Fig. 1: Broadband light curve obtained from the four independent reductions. Each colour indicates a different reduction. Data points at the original time sampling are shown as small open circles and a binning with 40 points per orbital period (170 integrations per bin, ∼30 minute sampling) is shown as filled circles, computed using the biweight_location function from astropy. Thin lines show the phase curve model. The 1σ uncertainties in each bin are obtained from the standard deviation of the residuals (data − model) divided by the square root of the number of points in that bin (170 integrations). The flux measured during the eclipse, which is the stellar flux only, is used as a reference and is shown as a dashed horizontal line.

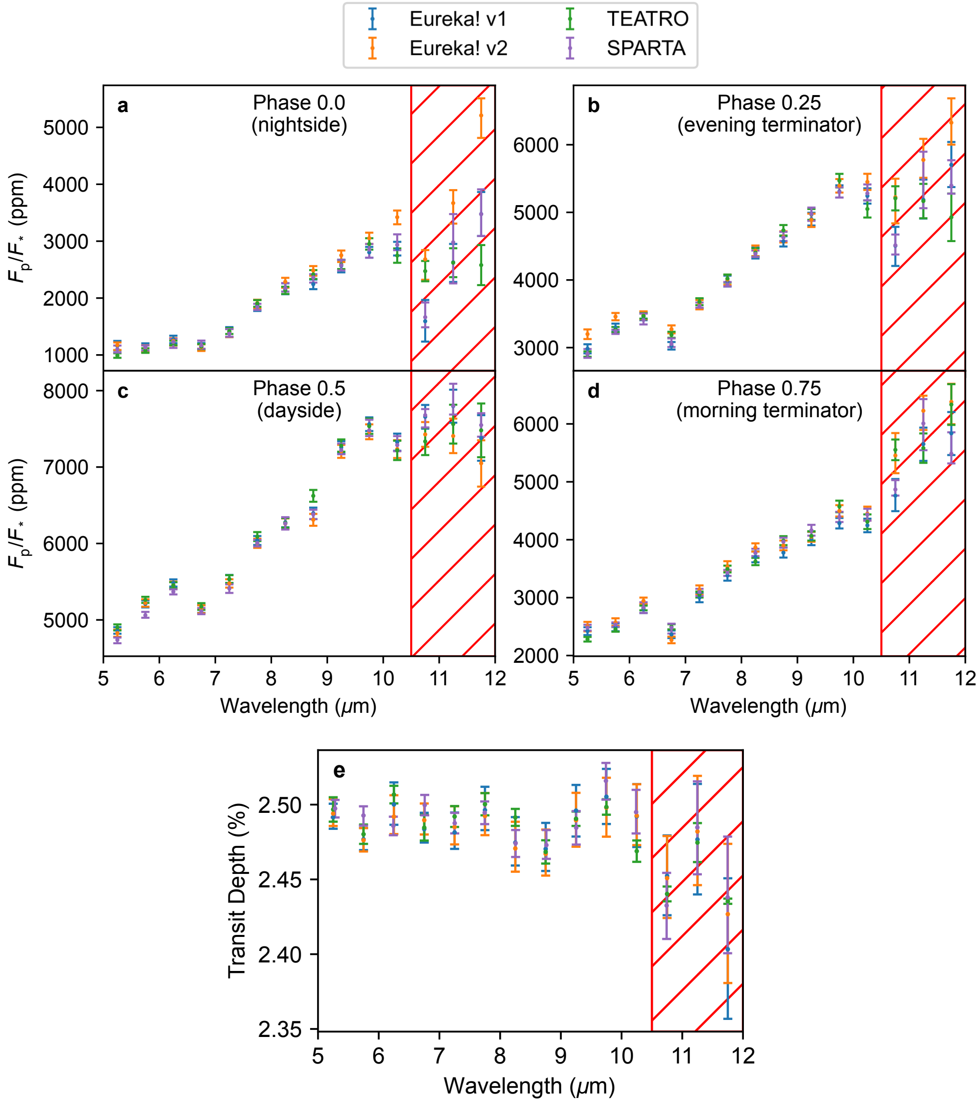

Supplementary Fig. 2: **Comparison of the phase-resolved and transmission spectra from different reductions.** Panels **a–d** show the phase resolved emission spectra from our four reductions with 1σ error bars, and panel **e** shows each of our transmission spectra with 1σ error bars. In general, there is good agreement about the phase-resolved spectra between our four semi-independent reductions. Larger differences arise >10.5 μm due to the "shadowed region effect" (indicated with red hatching). The transmission spectrum appears flat (within uncertainties) and shows no significant differences between reduction methods.
